# A faunistic revision of Fulgoromorpha and Cicadomorpha (Insecta, Hemiptera) of Lithuania

**DOI:** 10.3897/zookeys.1273.178263

**Published:** 2026-03-17

**Authors:** Oleg Borodin, Andrius Petrašiūnas, Emilija Sriubaite, Vytautas Tamutis, Villu Soon

**Affiliations:** 1 Institute of Ecology and Earth Science, University of Tartu, Tartu, Estonia Institute of Ecology and Earth Science, University of Tartu Tartu Estonia; 2 Institute of Biosciences, Vilnius University Life Sciences Center, Vilnius, Lithuania Natural History Museum, University of Tartu Tartu Estonia; 3 Kaunas Tadas Ivanauskas Museum of Zoology, Kaunas, Lithuania Institute of Biosciences, Vilnius University Life Sciences Center Vilnius Lithuania; 4 Natural History Museum, University of Tartu, Tartu, Estonia Kaunas Tadas Ivanauskas Museum of Zoology Kaunas Lithuania

**Keywords:** Auchenorrhyncha, catalogue, checklist, Lithuania, true hoppers

## Abstract

The article presents an updated checklist and a catalogue of Fulgoromorpha and Cicadomorpha of Lithuania based on analysis of published records, material preserved in available institutional and private collections of Latvia, Lithuania, and Estonia, and material collected between 2017 and 2025 from various regions of Lithuania. A total of 360 species belonging to 147 genera of Fulgoromorpha and Cicadomorpha are confirmed for the Lithuanian fauna; 20 species are reported in this article for the first time from Lithuania, four of which are newly recorded in the Baltic States.

## Introduction

Auchenorrhyncha comprises a large group of Hemiptera and is divided into two suborders: Fulgoromorpha and Cicadomorpha. According to the global catalogue of Auchenorrhyncha by Dmitriev et al. (2022), the world fauna comprises more than 46,600 species, divided into 35 families. The taxon has an almost worldwide distribution, occurring on all continents except Antarctica. The vast majority of Auchenorrhyncha are phytophagous, except for representatives of the family Achilidae, which are known to be mycophagous. Among Auchenorrhyncha, many species are pests of agricultural and ornamental plants, and many are highly specialised vectors of plant pathogens.

Despite a long history of studying Auchenorrhyncha, knowledge is unevenly distributed across different regions of the planet. There are major regional reviews of Auchenorrhyncha that enable us to draw conclusions about faunal changes over historical time, for example, by recording new species that were reliably absent before, or conversely by documenting the disappearance of particular species from specific areas. Examples of such regions include Central Europe (Holzinger et al. 2003) and Northern Europe (Söderman et al. 2009; Endrestøl 2013), individual countries of southern (Guglielmino et al. 2015) and eastern Europe (Borodin 2015a, 2015b; Tishechkin 2022), North America (Santos et al. 2024), Southeast Asia (Jiang et al. 2024), Australia, and Oceania (Evangelista de Souza et al. 2024). However, in all cases, gaps in the knowledge of particular local areas or taxonomic groups exist, and the authors themselves often highlight these. A similar situation is observed in Lithuania. The history of studying Auchenorrhyncha in the country spans more than 200 years, although with several exceptions, it would be hard to call it thorough.

Supposedly, the first published reference about Auchenorrhyncha from Lithuania is a work of B.A. Gimmerthal (1846), where *Ledra aurita* (Linnaeus, 1758) “from Kowno” (currently Kaunas; the specimen is deposited in the collection of the Institute of Biology, University of Latvia (**LUBI**)) is mentioned among other species from territories of current Latvia and Estonia. However, information about even older collections from Lithuania, dating back to approximately 1801–1802, is documented in the manuscripts of Karol de Perthées (Gębicki et al. 2017).

A century-long gap in research followed until the Estonian entomologist Juhan Vilbaste carried out extensive collecting of Auchenorrhyncha in the 1960s and compiled a list of 296 species from Lithuania (Vilbaste 1974). Following this work, only sporadic data were published by a few authors. Mensonienė (1979) listed 33 species from *Rosa* and *Aronia* plantations, four of which were new to Lithuania. After light-trapping in the Čepkeliai Nature Reserve, 13 new species were added by Söderman and Dapkus (2009), and 18 new species were discovered by sweep netting in the southeastern part of the country (Söderman and Rintala 2009), combining to the total of 331 species, listed for Lithuania in an annotated catalogue of the Auchenorrhyncha of Northern Europe (Söderman et al. 2009).

Some targeted research has also been conducted on Auchenorrhyncha as vectors of plant diseases (Staniulis and Genytė 1974; Genytė and Staniulis 1975, 1976; Ivanauskas et al. 2011, 2014a; Valiūnas et al. 2017), invasive species (Stalažs 2013), or protected species (Švitra 2007). Several recent works have added information on the distribution of species already known from Lithuania (Ivanauskas et al. 2014b; Petrašiūnas 2023), with two more species added to the list of those known from this country (Petrašiūnas 2021).

The primary objective of this work is to compile all available information on Lithuanian Auchenorrhyncha, including all known publications and collection data.

## Materials and methods

### Literature review

The most recent major regional synthesis on the Auchenorrhyncha of Lithuania is the catalogue of the Auchenorrhyncha of Northern Europe (Söderman et al. 2009), supplemented by several subsequent articles (e.g. Ivanauskas et al. 2011, 2014a, 2014b; Petrašiūnas 2021). The data from Lithuania included in this catalogue, as well as in the catalogue of European Auchenorrhyncha (Nast 1987), were derived primarily from the first regional review of the Auchenorrhyncha of Lithuania and Latvia prepared by the Estonian entomologist Juhan Vilbaste (1974). This work, in fact, remains the only synthesis explicitly devoted to the Lithuanian Auchenorrhyncha and was used in the present study as the primary source for assessing the state of knowledge of particular regions of Lithuania.

This article considers all synonyms that have been used in the literature reporting the occurrence of Auchenorrhyncha in Lithuania. This information is included in the annotated list provided below. In addition, relevant data from open biodiversity databases and platforms were used (primarily iNaturalist (2025)). Data from these platforms were used only for species recorded for the first time in Lithuania.

### New collection records

The data on Auchenorrhyncha were obtained during the implementation of various projects from 2017 to 2022, 2024, and 2025, where a broad range of methods was employed – not only sweeping with an entomological net, but also using Moericke, Malaise, NZI, and Barber pitfall traps. Material from light trapping on 21 August 2015 at Karmazinai, provided by E. Õnap, was also examined. All collected material was placed either on cotton layers or in alcohol, labelled appropriately and is deposited in the Natural History Museum, University of Tartu (**TUZ**) or Museum of Zoology, Vilnius University (**MZVU**) collections. Between 2023 and 2025, additional fieldwork was conducted in Lithuania. Supplementary sampling was conducted at more than 50 localities, and more than 2,000 Auchenorrhyncha specimens were collected. In total, 500 samples were processed, containing more than 14,000 Auchenorrhyncha specimens. In addition, materials deposited in the following collections were investigated:

**MZVU** Museum of Zoology, Vilnius University, Vilnius, Lithuania;

**KTIZM** Kaunas T. Ivanauskas Zoological Museum, Kaunas, Lithuania;

**NRC** State Scientific Research Institute Nature Research Centre, Vilnius, Lithuania;

**LUBI** Institute of Biology, University of Latvia, Riga, Latvia;

**TUZ** Natural History Museum, University of Tartu, Tartu, Estonia;

**IZBE** Entomological Collection of the Estonian University of Life Sciences, Tartu, Estonia.

In the Entomological Collection (LDM) of the Latvian National Museum of Natural History (Riga, Latvia) and the Collection of Invertebrates (DUBD) of Daugavpils University (Daugavpils, Latvia), no Auchenorrhyncha specimens from Lithuania were found.

The IZBE collection includes the material of J. Vilbaste, which formerly belonged to the Institute of Zoology and Botany of the Estonian Academy of Sciences. This collection served as the basis for the review of the Auchenorrhyncha of Lithuania and Latvia (Vilbaste 1974). However, J. Vilbaste’s article presented only summarised data, indicating geobotanical regions in which particular species were recorded. Detailed locality information was provided only for species known from fewer than 5–6 sites. In this regard, we included in the current article only those localities from this collection that were not explicitly mentioned in J. Vilbaste’s paper. In total, more than 3,170 Auchenorrhyncha specimens from Lithuania, housed in this collection, were analysed.

Below is a list of all localities used in this study. The localities are grouped by municipality. For each locality, the code of the municipality to which the locality belongs and a serial number are provided. In each municipality, the numbers are in ascending order. Localities from which only specimens from the J. Vilbaste collection are known are marked (V). Localities for which both specimens from the J. Vilbaste collection and additional data are available are marked (V+).

**Akmenė District Municipality**: Akm01 – Kamanų preserve, 56.2503°N, 22.7302°E.

**Alytus City Municipality**: AlytC01 – Alytus (1), 54.3815°N, 24.0174°E (V); AlytC02 – Alytus (2), 54.4183°N, 24.0708°E (V); AlytC03 – Alytus (3), 54.4208°N, 24.0489°E (V).

**Alytus District Municipality**: AlytD01 – Ąžuoliniai env. (1), 54.4337°N, 23.5838°E; AlytD02 – Ąžuoliniai env. (2), 54.4395°N, 23.5887°E; AlytD03 – Bundoriai, 54.5057°N, 24.0315°E (V); AlytD04 – Daugai env., 54.3601°N, 24.3452°E (V); AlytD05 – Punia env., 54.5101°N, 24.1063°E (V); AlytD06 – Punia forest, 54.5244°N, 24.0425°E; AlytD07 – Punios šilas, 54.5409°N, 24.0873°E; AlytD08 – Simnas env., 54.3836°N, 23.6469°E (V); AlytD09 – Žuvintas env., 54.4571°N, 23.6399°E (V+).

**Anykščiai District Municipality**: Anyk01 – Andrioniškis env., 55.5974°N, 25.0441°E (V); Anyk02 – Antanava env., 55.3970°N, 25.1232°E (V+); Anyk03 – Burbiškis env., 55.4976°N, 25.2215°E; Anyk04 – Inkūnai env., 55.6497°N, 25.1781°E; Anyk05 – Skaistis lake env., 55.6847°N, 25.2086°E; Anyk06 – Skvarbinis lake env., 55.6842°N, 25.2450°E; Anyk07 – Traupis env., 55.5124°N, 24.7566°E.

**Birštonas Municipality**: B01 – Birštonas, 54.6046°N, 24.0304°E (V).

**Biržai District Municipality**: Bir01 – Biržai env., 56.2020°N, 24.7560°E (V); Bir02 – Kilučių lake shore, 56.1835°N, 24.7650°E; Bir03 – Ločiai env., 56.3776°N, 24.8586°E; Bir04 – Paroveja, 56.2452°N, 24.8604°E (V).

**Druskininkai Municipality**: Drus01 – Druskininkai env., 54.0049°N, 23.9864°E (V+); Drus02 – Margai env., 54.1497°N, 23.9356°E; Drus03 – Miškas Beržalapis, 53.9667°N, 23.9704°E; Drus04 – Miškas Paravė, 54.0489°N, 24.1190°E; Drus05 – Raigardas forest, 53.9676°N, 24.0357°E (V); Drus06 – Ratnyčia env, 54.0014°N, 24.0189°E.

**Elektrėnai Municipality**: El01 – Bražuolė campsite, 54.7558°N, 24.9571°E; El02 – Paneriai env., 54.7833°N, 24.9087°E (V); El03 – Panerių 1 Miškas forest, 54.7767°N, 24.9361°E; El04 – Zabarija env., 54.8047°N, 24.8630°E.

**Ignalina District Municipality**: Ign01 – Ažvinčių preserve, 55.4340°N, 26.0474°E; Ign02 – Beniūnai env., 55.5202°N, 26.4963°E; Ign03 – Ceikiniai Quarry, 55.2590°N, 26.2763°E; Ign04 – Dringis env., 55.3737°N, 26.1117°E (V); Ign05 – Ignalina env. (1), 55.3284°N, 26.1537°E; Ign06 – Ignalina env. (2), 55.3385°N, 26.1645°E (V); Ign07 – Kazimieriškė env., 55.2874°N, 26.0225°E; Ign08 – Kazokinė env., 55.2853°N, 26.1847°E; Ign09 – Nagėnai env., 55.5024°N, 26.4619°E; Ign10 – Palūšė env., 55.3281°N, 26.1014°E (V).

**Jonava District Municipality**: Jona01 – Jonava env., 55.0727°N, 24.2795°E (V+).

**Jurbarkas District Municipality**: Jurb01 – Jurbarkas env., 55.0768°N, 22.7617°E; Jurb02 – Seredžius env., 55.0814°N, 23.4186°E (V); Jurb03 – Smukučiai env., 55.0847°N, 22.6822°E; Jurb04 – Viešvilė env. (1), 55.0556°N, 22.3879°E; Jurb05 – Viešvilė env. (2), 55.0610°N, 22.3878°E; Jurb06 – Viešvilė env. (3), 55.0954°N, 22.4088°E; Jurb07 – Viešvilė preserve, Smaladaržis env., 55.1163°N, 22.4406°E.

**Kaišiadorys District Municipality**: Kaiš01 – Girelė env., 54.8610°N, 24.3850°E; Kaiš02 – Kaišiadorys city, 54.8652°N, 24.4543°E; Kaiš03 – Rumšiškės env., 54.8684°N, 24.2117°E; Kaiš04 – Strošiūnų forest, 54.8334°N, 24.4856°E; Kaiš05 – Vadų Miškas forest, 54.9559°N, 24.3377°E; Kaiš06 – Vaiguvos env., 54.7869°N, 24.2120°E; Kaiš07 – Žiežmariai env., 54.8052°N, 24.4390°E (V).

**Kalvarija Municipality**: Kal01 – Juodeliai env., 54.3942°N, 23.1313°E; Kal02 – Kalvarija env., 54.4144°N, 23.2270°E (V+); Kal03 – Piliakalniai env., 54.3966°N, 23.0917°E (V).

**Kaunas City Municipality**: KauC01 – Dainava env., 54.9133°N, 23.9683°E; KauC02 – Kaunas Botanical garden, 54.8686°N, 23.9072°E; KauC03 – Panemunė Jiesios (1), 54.8503°N, 23.9378°E; KauC04 – Panemunė Jiesios (2), 54.8514°N, 23.9404°E; KauC05 – Panemunė Jiesios (3), 54.8515°N, 23.9400°E; KauC06 – Panemunė Jiesios (4), 54.8531°N, 23.9383°E; KauC07 – Panemunė Jiesios (5), 54.8547°N, 23.9376°E; KauC08 – Panemunė Jiesios (6), 54.8570°N, 23.9404°E; KauC09 – Petrašiūnai env., 54.8797°N, 24.0076°E.

**Kaunas District Municipality**: KauD01 – Babtai env., 55.0874°N, 23.8009°E (V); KauD02 – Braziūkai env. (1), 54.9014°N, 23.4847°E; KauD03 – Braziūkai env. (2), 54.9021°N, 23.4841°E; KauD04 – Braziūkai env. (3), 54.9081°N, 23.4820°E; KauD05 – Dubravos forest, 54.8506°N, 24.0635°E; KauD06 – Garliava env. (1), 54.8273°N, 23.8760°E; KauD07 – Garliava env. (2), 54.8100°N, 23.9038°E; KauD08 – Girionys env., 54.8544°N, 24.0387°E; KauD09 – Kačerginė env., 54.9341°N, 23.7123°E; KauD10 – Kamšos forest, 54.9004°N, 23.7925°E; KauD11 – Karalgirio forest, 55.1189°N, 23.6565°E; KauD12 – Karmėlava env., 54.9656°N, 24.0716°E; KauD13 – Kazliškiai env., 54.8823°N, 23.8554°E; KauD14 – Muniškiai env., 55.0728°N, 23.8258°E; KauD15 – Netoniai env., 54.9399°N, 23.7253°E; KauD16 – Noreikiškės env., 54.8885°N, 23.8548°E; KauD17 – Pakarklė forest, 55.0142°N, 23.6001°E; KauD18 – Ringaudai env., 54.8910°N, 23.7951°E; KauD19 – Ringovė entomological reserve, 55.0503°N, 23.5184°E; KauD20 – Senieji Bernatoniai (1), 54.9860°N, 23.7689°E; KauD21 – Senieji Bernatoniai (2), 54.9884°N, 23.7753°E; KauD22 – Senieji Bernatoniai (3), 54.9898°N, 23.7744°E; KauD23 – Šlienava env., 54.8656°N, 24.0916°E; KauD24 – Viršužiglis env., 54.8275°N, 24.1866°E.

**Kazlų Rūda Municipality**: K.R.01 – Ąžuolų Būda env., 54.7015°N, 23.5213°E (V); K.R.02 – Braziūkų forest, 54.8942°N, 23.4720°E; K.R.03 – Jūrė env., 54.7595°N, 23.5894°E; K.R.04 – Kajackai env., 54.8045°N, 23.5877°E.

**Kėdainiai District Municipality**: Kėd01 – Kėdainiai env., 55.2882°N, 23.9716°E (V+); Kėd02 – Vincentava env., 55.1916°N, 23.6632°E.

**Kelmė District Municipality**: Kel01 – Beržėnai env., 55.8256°N, 22.8287°E; Kel02 – Galvydiškė env., 55.7822°N, 22.9742°E; Kel03 – Gudmoniškė env., Alkaline fen (1), 55.8710°N, 22.9392°E; Kel04 –Alkaline fen (2), 55.8710°N, 22.9425°E; Kel05 –Alkaline fen (3), 55.8713°N, 22.9427°E.

**Klaipėda City Municipality**: KlaiC01 – Klaipėda env., 55.7033°N, 21.1443°E (V); KlaiC02 – Melnragė I, 55.7328°N, 21.0872°E; KlaiC03 – Melnragė II, 55.7539°N, 21.0833°E.

**Klaipėda District Municipality**: KlaiD01 – Čiobrelių rojus, 55.6194°N, 21.4105°E; KlaiD02 – Dercekliai env., 55.5645°N, 21.2490°E; KlaiD03 – Dovilai env., 55.6836°N, 21.3661°E (V); KlaiD04 – Gargždai env., 55.7117°N, 21.3952°E; KlaiD05 – Pajūrio RP Šaipiai, 55.8576°N, 21.0870°E; KlaiD06 – Šaipiai env., 55.8444°N, 21.0652°E; KlaiD07 – Tyrai Klišiai env., 55.5320°N, 21.2264°E.

**Kretinga District Municipality**: Kre01 – Darbėnai env., 56.0267°N, 21.2336°E; Kre02 – Kartena env., 55.9171°N, 21.4752°E.

**Kupiškis District Municipality**: Kup01 – Bugailiškiai env. (1), 55.7538°N, 25.2426°E; Kup02 – Bugailiškiai env. (2), 55.7540°N, 25.2493°E; Kup03 – Juodpėnai env., 55.8032°N, 25.2187°E; Kup04 – Puponys env., 55.8019°N, 25.1178°E; Kup05 – Šileikiai env., 55.8053°N, 25.2232°E.

**Lazdijai District Municipality**: Laz01 – Avižieniai env., 54.1836°N, 23.7291°E; Laz02 – Meteliai env., 54.3003°N, 23.7405°E (V); Laz03 – Miškas Bijotai forest (1), 54.2778°N, 23.7564°E; Laz04 – Miškas Bijotai forest (2), 54.2851°N, 23.7461°E; Laz05 – Seirijai env., 54.2349°N, 23.8166°E (V); Laz06 – Veisiejai env., 54.1023°N, 23.6945°E (V).

**Marijampolė Municipality**: Mar01 – Marijampolė env., 54.5590°N, 23.3488°E (V+).

**Mažeikiai District Municipality**: Maž01 – Pušinė env., 56.1114°N, 22.1459°E; Maž02 – Varduva env., 56.3168°N, 22.2925°E (V).

**Molėtai District Municipality**: Mol01 – Aluntas env., 55.3503°N, 25.2930°E (V); Mol02 – Giraičiai env., 55.0486°N, 25.5077°E (V); Mol03 – Molėtai env., 55.2286°N, 25.4146°E (V); Mol04 – Skinderiškė env., 55.2395°N, 25.6262°E; Mol05 – Žagarai env., 55.2455°N, 25.6322°E.

**Neringa Municipality**: Ner01 – Curonian Split, 55.3301°N, 21.0175°E (V); Ner02 – Juodkrantė env. (1), 55.5238°N, 21.1031°E; Ner03 – Juodkrantė env. (2), 55.5339°N, 21.1002°E; Ner04 – Juodkrantė env. (3), 55.5390°N, 21.1147°E (V); Ner05 – Juodkrantė env. (4), 55.5460°N, 21.1207°E; Ner06 – Juodkrantė env. (5), 55.5551°N, 21.1216°E; Ner07 – Nida env., 55.4369°N, 21.0724°E (V); Ner08 – Pervalka env. (1), 55.4026°N, 21.0916°E; Ner09 – Pervalka env. (2), 55.4028°N, 21.0852°E.

**Pagėgiai Municipality**: Pag01 – Pagėgiai env., 55.1490°N, 21.8823°E

**Palanga City Municipality**: PalC01 – Palanga, 55.9202°N, 21.0678°E

**Palanga Municipality**: PalM01 – Būtingės Miškas forest, 56.0717°N, 21.1211°E; PalM02 – Monciškė env., 56.0004°N, 21.0728°E; PalM03 – Šventoji env., 56.0274°N, 21.0809°E; PalM04 – Šventoji River, 55.0853°N, 24.3999°E.

**Panevėžys City Municipality**: PanC01 – Panevėžys env., 55.7353°N, 24.3617°E (V+)

**Panevėžys District Municipality**: PanD01 – Aukštadvaris env., 55.4788°N, 24.2986°E (V); PanD02 – Gegužinė env., 55.8491°N, 24.3437°E (V); PanD03 – Giniūnai env., 55.7670°N, 24.0464°E (V); PanD04 – Piniava env., 55.7799°N, 24.3618°E (V); PanD05 – Ramygala env., 55.5088°N, 24.3033°E (V).

**Pasvalys District Municipality**: Pas01 – Talačkoniai env., 56.0269°N, 24.3629°E (V).

**Plungė District Municipality**: Plu01 – Miškas Plokštinė (1), 56.0119°N, 21.8678°E; Plu02 – Miškas Plokštinė (2), 56.0264°N, 21.9049°E; Plu03 – Plateliai env., 56.0430°N, 21.8164°E (V); Plu04 – Stalgėnai env., 55.8231°N, 21.8870°E (V).

**Prienai District Municipality**: Prie01 – Balbieriško forest, 54.5157°N, 23.8272°E; Prie02 – Karmėlava forest, 54.6304°N, 24.1122°E; Prie03 – Norkūnai env. (1), 54.5007°N, 23.9460°E; Prie04 – Norkūnai env. (2), 54.5010°N, 23.9415°E; Prie05 – Norkūnai env. (3), 54.5013°N, 23.9420°E; Prie06 – Prienų šilas forest (1), 54.6000°N, 23.9250°E; Prie07 – Prienų šilas forest (2), 54.5982°N, 23.9240°E.

**Radviliškis District Municipality**: Rad01 – Burūnai env., 55.5266°N, 23.5592°E; Rad02 – Debeikiai env. (1), 55.6089°N, 23.3460°E; Rad03 – Debeikiai env. (2), 55.6075°N, 23.3435°E; Rad04 – Papušinis env., 55.5585°N, 23.3854°E (V); Rad05 – Radviliškis env., 55.8108°N, 23.5762°E; Rad06 – Verduliai env., 55.7708°N, 23.6287°E.

**Raseiniai District Municipality**: Ras01 – Ariogala env., 55.2604°N, 23.4728°E (V); Ras02 – Kaulakiai env., 55.4299°N, 23.2560°E (V+); Ras03 – Plikiai env., 55.2065°N, 23.5305°E; Ras04 – Saudininkai env., 55.2563°N, 23.4796°E (V); Ras05 – Žaiginys env., 55.4927°N, 23.3372°E (V).

**Rietavas Municipality**: Rie01 – Tverai env., 55.7331°N, 22.1460°E (V)

**Rokiškis District Municipality**: Rok01 – Andrikava env., 55.8997°N, 25.8188°E; Rok02 – Bargailių forest, 55.5482°N, 23.4908°E; Rok03 – Bradesiai env., 55.8274°N, 25.8953°E; Rok04 – Kačergiškis env., 55.9101°N, 25.8304°E; Rok05 – Mičiūnai env., 55.8819°N, 25.8362°E; Rok06 – Pasarčiai env., 55.8524°N, 25.8707°E; Rok07 – Praviršulio tyrelis reserve, 55.5219°N, 23.4528°E; Rok08 – Rokiškis env., 55.9629°N, 25.5683°E; Rok09 – Rudžionys env., 55.9141°N, 25.8114°E.

**Šakiai District Municipality**: Šak01 – Juškinės forest (1), 55.0199°N, 23.4441°E; Šak02 – Juškinės forest (2), 55.0139°N, 23.4623°E; Šak03 – Lekėčiai env., 54.9823°N, 23.4940°E; Šak04 – Pavilkijys env., 55.0352°N, 23.5612°E; Šak05 – Tervydoniai env. (01), 55.0276°N, 23.4428°E; Šak06 – Tervydoniai env. (02), 55.0279°N, 23.4441°E; Šak07 – Tervydoniai env. (03), 55.0168°N, 23.4491°E; Šak08 – Tervydoniai env. (04), 55.0270°N, 23.4469°E; Šak09 – Tervydoniai env. (05), 55.5540°N, 22.4481°E; Šak10 – Tervydoniai env. (06), 55.0289°N, 23.4447°E; Šak11 – Tervydoniai env. (07), 55.0280°N, 23.4492°E; Šak12 – Tervydoniai env. (08), 55.0274°N, 23.4479°E; Šak13 – Tervydoniai env. (09), 55.0273°N, 23.4487°E; Šak14 – Tervydoniai env. (10), 55.0272°N, 23.4433°E; Šak15 – Tervydoniai env. (11), 55.0276°N, 23.4450°E; Šak16 – Tervydoniai env. (12), 55.0274°N, 23.4486°E; Šak17 – Vidušilio Miškas forest, 55.0117°N, 23.3931°E.

**Šalčininkai District Municipality**: Šal01 – Stakai env., 54.2977°N, 25.5346°E; Šal02 – Zajašiškės env., 54.1865°N, 25.5654°E.

**Šiauliai District Municipality**: Šiau01 – Agailių forest, 56.1172°N, 22.9668°E; Šiau02 – Bubiai env., 55.8734°N, 23.1423°E; Šiau03 – Bulėnų pelkė bog (1), 55.8721°N, 23.0461°E; Šiau04 – Bulėnų pelkė bog (2), 55.8727°N, 23.0459°E; Šiau05 – Dzidai env., 55.8371°N, 23.1707°E; Šiau06 – Slydžiai env., 55.8326°N, 23.1580°E; Šiau07 – Visdergiai, Alkaline fen, 55.9174°N, 22.9028°E.

**Šilalė District Municipality**: Šila01 – Kaltinėnai env., 55.5640°N, 22.4485°E (V).

**Šilutė District Municipality**: Šilu01 – Degučiai env., 55.3201°N, 21.7615°E (V); Šilu02 – Inkakliai env., 55.4821°N, 21.5789°E (V); Šilu03 – Medžioklės pelkė bog, 55.2560°N, 21.4557°E; Šilu04 – Rupkalviai env. (1), 55.3168°N, 21.4076°E; Šilu05 – Rupkalviai env. (2), 55.3189°N, 21.4212°E; Šilu06 – Rupkalvių upland bog (1), 55.2957°N, 21.4488°E; Šilu07 – Rupkalvių upland bog (2), 55.2959°N, 21.4513°E; Šilu08 – Rupkalvių upland bog (3), 55.2962°N, 21.4497°E; Šilu09 – Rusnė env., 55.2990°N, 21.3774°E (V); Šilu10 – Šilmeižiai env., Šyša env. (1), 55.3127°N, 21.4050°E; Šilu11 – Šilmeižiai env., Šyša env. (2), 55.3147°N, 21.4048°E; Šilu12 – Šilmeižiai env., Šyša env. (3), 55.3197°N, 21.4063°E; Šilu13 – Ventė Cape, 55.3411°N, 21.1899°E; Šilu14 – Žalgiriai env., 55.2953°N, 21.4528°E.

**Širvintos District Municipality**: Širv01 – Dūdai env., 55.0958°N, 24.7568°E; Širv02 – Pajuodžiai env., 54.8845°N, 24.9591°E.

**Švenčionys District Municipality**: Šven01 – Aibutiškis lake, 55.2478°N, 25.8099°E; Šven02 – Antaliedė env., 55.2225°N, 25.9239°E; Šven03 – Asveja env., 54.9946°N, 25.6137°E (V); Šven04 – Augustavas env., 55.0025°N, 26.0019°E; Šven05 – Baranava Miškas forest, 55.1834°N, 25.9615°E; Šven06 – Bedugnis lake, 55.2509°N, 25.8141°E; Šven07 – Beržuvis env., 55.1710°N, 26.1903°E; Šven08 – Didžioji Bala, 55.2337°N, 25.7472°E; Šven09 – Jusiai env. (1), 55.2003°N, 25.9542°E; Šven10 – Jusiai env. (2), 55.1980°N, 25.9643°E; Šven11 – Kaltanėnai env. (1), 55.2486°N, 26.0078°E; Šven12 – Kaltanėnai env. (2), 55.2487°N, 26.0099°E; Šven13 – Kaltanėnai env. (3), 55.2488°N, 26.0045°E; Šven14 – Labanoras regional park (1), 55.2672°N, 25.7740°E (V); Šven15 – Labanoras regional park (2), 55.2226°N, 25.7408°E; Šven16 – Labanoras regional park (3), 55.2319°N, 25.7519°E; Šven17 – Labanoras regional park (4), 55.2347°N, 25.6843°E; Šven18 – Labanoras regional park (5), 55.2348°N, 25.6832°E; Šven19 – Labanoras regional park (6), 55.2358°N, 25.6843°E; Šven20 – Labanoras regional park (7), 55.2663°N, 25.7936°E; Šven21 – Liūlinė env., 55.1036°N, 25.9553°E; Šven22 – Malinovka env., 55.0525°N, 26.0225°E; Šven23 – Noselėnai env., 54.9062°N, 25.6921°E; Šven24 – Pabradė env., 54.9830°N, 25.7649°E (V); Šven25 – Paibutiškis env. (1), 55.2468°N, 25.8026°E; Šven26 – Paibutiškis env. (2), 55.2468°N, 25.8047°E; Šven27 – Paibutiškis env. (3), 55.2472°N, 25.8104°E; Šven28 – Paminėlė env. (1), 55.2466°N, 25.7532°E; Šven29 – Paminėlė env. (2), 55.2471°N, 25.7502°E; Šven30 – Paminėlė env. (3), 55.2474°N, 25.7479°E; Šven31 – Pašekštis env., 55.2422°N, 25.8119°E; Šven32 – Platumai env., 55.1714°N, 25.9800°E; Šven33 – Šipiniškis env., 55.2639°N, 25.7531°E.

**Tauragė District Municipality**: Tau01 – Buveinių lake env., 55.1823°N, 22.4387°E; Tau02 – Draudeniai env., 55.3088°N, 21.9933°E (V); Tau03 – Tauragė env., 55.2546°N, 22.2892°E (V); Tau04 – Viešvilė preserve (1), 55.1448°N, 22.4150°E; Tau05 – Viešvilė preserve (2), 55.1794°N, 22.4629°E.

**Telšiai District Municipality**: Telš01 – Didžiųjų Burbiškių mound, 55.7636°N, 22.5011°E; Telš02 – Lukšto env., 55.7146°N, 22.3339°E (V); Telš03 – Telšiai env., 55.9839°N, 22.2503°E (V); Telš04 – Užmarkija env., 56.1115°N, 22.1789°E; Telš05 – Varniai env., 55.7423°N, 22.3717°E.

**Trakai District Municipality**: Trak01 – Akmena env., 54.6517°N, 24.9022°E; Trak02 – Bražuolė env., 54.6692°N, 24.9003°E; Trak03 – Būda env., 54.7096°N, 24.9518°E (V); Trak04 – Dėdeliškės env., 54.6683°N, 25.0192°E; Trak05 – Jovariškės env., 54.6508°N, 24.8683°E; Trak06 – Kariotiškės env., 54.6525°N, 25.0667°E; Trak07 – Kirtimai env., 54.6333°N, 24.5511°E; Trak08 – Lake Skaistis env. I, 54.6833°N, 24.9525°E; Trak09 – Narezkai env., 54.6258°N, 24.9796°E; Trak10 – Pagelužys env., 54.4399°N, 24.7914°E; Trak11 – Pakrempė env. (1), 54.4925°N, 25.1130°E; Trak12 – Pakrempė env. (2), 54.4828°N, 25.1153°E; Trak13 – Pakrempė env. (3), 54.5093°N, 25.1231°E; Trak14 – Pakrempė env. (4), 54.5172°N, 25.1562°E; Trak15 – Paluknis env., 54.5009°N, 24.9848°E (V); Trak16 – Ropėjos Miškas forest, 54.5333°N, 25.0167°E; Trak17 – Strėva env. (1), 54.4999°N, 25.1374°E (V+); Trak18 – Strėva env. (2), 54.5854°N, 24.7050°E (V+); Trak19 – Trakų Vokė, 54.6025°N, 25.0350°E.

**Utena District Municipality**: Ute01 – Aišetos env., 55.3086°N, 25.7371°E (V); Ute02 – Minčios env., 55.4871°N, 25.9793°E; Ute03 – Paluknis env., 55.4254°N, 25.3912°E; Ute04 – Utena env., 55.5035°N, 25.5988°E; Ute05 – Vyžuonos env., 55.5730°N, 25.4881°E; Ute06 – Vyžuonų Miškas forest (1), 55.5894°N, 25.5283°E; Ute07 – Vyžuonų Miškas forest (2), 55.5933°N, 25.5469°E.

**Varėna District Municipality**: Var01 – Čepkelių raistas marsh, 54.0110°N, 24.4929°E; Var02 – Dubininkas env., 54.0968°N, 24.2850°E; Var03 – Forest Luciškė, 54.0823°N, 24.3824°E; Var04 – Grybaulia env. (1), 53.9900°N, 24.3628°E; Var05 – Grybaulia env. (2), 54.0030°N, 24.3818°E; Var06 – Gudakiemis env., 54.4353°N, 24.9842°E; Var07 – Gudaraistis env., 54.2336°N, 24.1747°E; Var08 – Ilgininkai env., 54.2563°N, 24.1620°E (V); Var09 – Jablanavo Miškas forest, 54.1374°N, 24.2118°E; Var10 – Jonionys env., 54.1520°N, 24.1342°E; Var11 – Krokšlio Miškas forest, 54.0472°N, 24.6413°E; Var12 – Lynelis env., 54.3535°N, 24.4451°E (V); Var13 – Marcinkonys env., Barsukinės forest, 54.0368°N, 24.4040°E; Var14 – Mardasavas env. (1), 54.1385°N, 24.3193°E; Var15 – Mardasavas env. (2), 54.1509°N, 24.3183°E; Var16 – Matuizos env., 54.2744°N, 24.6918°E; Var17 – Merkinė env., 54.1644°N, 24.1854°E (V); Var18 – Miškas Diniškės forest, 54.0886°N, 24.1966°E; Var19 – Musteika env., 53.9575°N, 24.3706°E; Var20 – Musteika, Aukštakalnio forest, 53.9384°N, 24.3916°E; Var21 – Naujieji Valkininkai (1), 54.3486°N, 24.7412°E; Var22 – Naujieji Valkininkai (2), 54.3524°N, 24.7243°E; Var23 – Pagarenda env., 53.9363°N, 24.4811°E; Var24 – Palkabalio Miškas forest, 54.1499°N, 24.5999°E; Var25 – Pamerkio Miškas forest, 54.2730°N, 24.5851°E; Var26 – Pirčiupiai env., 54.3991°N, 24.9549°E; Var27 – Pučkornių Miškas forest, 54.3489°N, 24.7453°E; Var28 – Puvočiai env. (1), 54.1140°N, 24.3050°E; Var29 – Puvočiai env. (2), 54.1193°N, 24.2973°E; Var30 – Puvočiai env. (3), 54.1129°N, 24.2989°E; Var31 – Puvočiai env. (4), 54.1138°N, 24.3126°E; Var32 – Puvočiai env. (5), 54.1144°N, 24.3084°E; Var33 – Skersabalės Miškas forest, 54.0917°N, 24.3769°E; Var34 – Subartonių forest, 54.1921°N, 24.1841°E; Var35 – Užuožerės forest, 54.0190°N, 24.4127°E; Var36 – Valkininkai env., 54.3602°N, 24.8377°E (V); Var37 – Varėna env., 54.2124°N, 24.5722°E (V); Var38 – Varėnos 2 Miškas forest, 54.1779°N, 24.5980°E; Var39 – Vėžionių Miškas forest, 54.3621°N, 24.4240°E; Var40 – Žeimiai env., 54.0817°N, 24.1079°E; Var41 – Žiūrai env., 54.1437°N, 24.4004°E.

**Vilkaviškis District Municipality**: Vilk01 – Alvitas env., 54.6353°N, 22.9238°E (V); Vilk02 – Virbalgiris forest, 54.6073°N, 22.7933°E.

**Vilnius City Municipality**: VilnC01 – Baniškės env., 54.7511°N, 25.4258°E; VilnC02 – Dravčionys env., 54.7283°N, 25.3742°E; VilnC03 – Guraičiai env., 54.7194°N, 25.3385°E; VilnC04 – Pagubė env., 54.8032°N, 25.3187°E; VilnC05 – Strapa env., 54.8683°N, 25.5593°E; VilnC06 – Verkiai env. (1), 54.7564°N, 25.3039°E; VilnC07 – Verkiai env. (2), 54.7519°N, 25.2955°E (V); VilnC08 – Vilnius (1), 54.6825°N, 25.3081°E; VilnC09 – Vilnius (2), 54.6872°N, 25.2797°E; VilnC10 – Vilnius (3), 54.7025°N, 25.2661°E; VilnC11 – Vilnius (4), 54.7406°N, 25.2714°E; VilnC12 – Vilnius (5), 54.7414°N, 25.2361°E; VilnC13 – Žirmūnai env., 54.7113°N, 25.2987°E.

**Vilnius District Municipality**: VilnD01 – Airėnai II env. (1), 54.8281°N, 24.9027°E; VilnD02 – Airėnai II env. (2), 54.8439°N, 24.9068°E; VilnD03 – Dvarčionys env., 54.8253°N, 25.4422°E; VilnD04 – Karmazinai env., 54.8150°N, 24.9440°E; VilnD05 – Pagiriai env., 54.5850°N, 25.1923°E (V); VilnD06 – Mažieji Gulbinai, 54.8228°N, 25.4397°E; VilnD07 – Nemenčinė env., 54.8480°N, 25.4678°E (V); VilnD08 – Raudonoji Bala, 54.8541°N, 25.2315°E; VilnD09 – Rudausiai env., 54.9087°N, 25.5922°E; VilnD10 – Rusausiai env., 54.9090°N, 25.5931°E; VilnD11 – Stirniai env., 54.7346°N, 25.0474°E (V).

**Zarasai District Municipality**: Zar01 – Antazavė env., 55.8111°N, 25.9232°E (V+); Zar02 – Pakačinės env., 55.8205°N, 25.8653°E; Zar03 – Salakas env., 55.5802°N, 26.1334°E (V); Zar04 – Zarasai env., 55.7318°N, 26.2457°E (V).

### Identification

Species-level identification was carried out only for adult specimens. Nymphs were also collected, but their species identity was not determined. When necessary, specimens were mounted on entomological pins. If preparations were required, primarily for genitalia investigations, dissections were performed. For this purpose, the abdomen from dry specimens was detached, placed in a ceramic crucible containing a 10% solution of KOH or NaOH, and heated over the flame of an alcohol burner for 20–30 seconds. Afterwards, the dissection was carried out in a drop of glycerine. Various models of stereoscopic microscopes were used for the work, predominantly Leica A60, Leica S9E and Leica S8APO. Subsequently, prepared material was mounted into a droplet of Euparal or Solakryl BMX (40% solution of synthetic resin in xylene) and pinned beneath the corresponding specimen. In total, more than 3,800 specimens were mounted, predominantly males.

Species identification was carried out using relevant identification keys (Le Quesne 1960, 1969; Ossiannilsson 1978, 1981, 1983; Le Quesne and Payne 1981; Anufriev and Emeljanov 1988; Holzinger et al. 2003). In some instances, specific literature on individual taxa was also used. The nomenclature and names of taxa used in this study follow those in the World Auchenorrhyncha Database (Dmitriev et al. 2022). All accumulated information, including both published and primary data, was entered into a database on the PlutoF platform (Abarenkov et al. 2010).

## Results

More than 18,900 Auchenorrhyncha specimens were examined. In total, we identified 360 species belonging to 147 genera. A checklist of all species currently recorded in Lithuania is provided below. The list includes all species recorded in Lithuania. For each species, a list of publications in which the species was reported from Lithuania is provided. Where distributional data are given in a publication, this information is also included and accompanied by the corresponding reference. The list also includes previously unpublished data from the collection of J. Vilbaste. Records from iNaturalist are accompanied by the corresponding observation number, which is also provided in parentheses after the respective record. Species newly recorded in Lithuania are marked with an asterisk (*), and those newly recorded in the Baltic States – with a double asterisk (**). Detailed information is provided on these records, along with additional comments.

### Fulgoromorpha Evans, 1946

**Delphacoidea Leach, 1815**

**Cixiidae Spinola, 1839**

**Cixiinae Spinola, 1839**

**Cixiini Spinola, 1839**

#### *Cixius* Latreille, 1804

***Cixius cunicularia* (Linnaeus, 1767)**: Vilbaste (1974); Nast (1987); Söderman et al. (2009). AlytD01; AlytD05; AlytD07; Ign09; KauC06; Plu04; Šak07; Ute01; Var34; VilnD11.

***Cixius distinguendus* Kirschbaum, 1868**: Vilbaste (1974); Nast (1987); Söderman et al. (2009). Šak07; Šak12; Šak15; Šak16; Šven01; Tau04; Var20; Var28; Zar03.

***Cixius nervosus* (Linnaeus, 1758)**: Vilbaste (1974); Nast (1987); Söderman et al. (2009). PanC01 (Mensonienė 1979). AlytD02; Bir01; Bir04; Kaiš06; KauC02; KauD05; KauD12; Mar01; Pas01; Rad01; Šak01; Šak05; Šak07; Šak16; Šiau01; Šilu02; Var28; VilnC01; VilnD07.

***Cixius similis* Kirschbaum, 1868**: Vilbaste (1974); Nast (1987); Söderman et al. (2009); Trivellone (2010). AlytD08; AlytD09; El01; Ign09; Šven15; Šven23; VilnD08.

#### *Tachycixius* Wagner, 1939

***Tachycixius pilosus* (Olivier, 1792)**: Nast (1987); Demir (2008); Söderman et al. (2009). Šilu02 (Vilbaste 1974). Bir01; Šven03.

### Pentastirini Emeljanov, 1971

#### *Pentastiridius* Kirschbaum, 1868

***Pentastiridius leporinus* (Linnaeus, 1761)** (= *Oliarus leporinus* (L., 1761)): Nast (1987); Söderman et al. (2009). VilnC09 (Vilbaste 1974). AlytD02; Ign09; KlaiD07; Šiau02; Trak13.

### Delphacidae Leach, 1815

**Asiracinae Motschulsky, 1863**

**Asiracini Motschulsky, 1863**

#### *Asiraca* Latreille, 1797

*Asiraca clavicornis (Fabricius, 1794) – Šal02: 1♀, 08 Jun. 2019, V. Tamutis leg.; Trak10: 1♂, 14 Aug. 2021, R. Markevičiūtė leg.; Var28: 1♀ (MZVUHe04111), 08 Aug. 2023, U. Paliukėnas leg.; VilnD09: 1♀, 15 Sept. 2024, A. Petrašiūnas leg.

iNaturalist records: Anyk07, 20 Aug. 2025 (iN272136560); Drus06, 27 May 2023 (iN163958009); El04, 04 Jul. 2022 (iN120658906); Ign05, 04 Jul. 2023 (iN166188981); KauD03, 01 May 2024 (iN212846796); KauD06, 29 Apr. 2023 (iN157492948); KauD23, 26 Aug. 2025 (iN273922326); Laz01, 27 Aug. 2025 (iN309657954); Prie03, 19 Jul. 2022 (iN122456379); Prie04, 05 Aug. 2023 (iN176744151); Prie05, 20 May 2023 (iN162729370); VilnC02, 01 Apr. 2025 (iN270993685); VilnC03, 02 May 2023 (iN159119259); VilnC04, 09 Sept. 2021 (iN94204293); VilnC08, 20 May 2023 (iN162821471); VilnC11, 24 Sept. 2020 (iN60605183); VilnC12, 19 Apr. 2024 (iN219271956); VilnD01, 30 Oct. 2023 (iN189588438); VilnD02, 10 Oct. 2021 (iN99483442); VilnD09, 15 Sept. 2024 (iN242514212); VilnD10, 17 May 2025 (iN282021475).

**Remarks**. The occurrence of the species in the Baltic States has long been considered doubtful. This was first noted by J. Vilbaste (1974), who referred to the works of B.A. Gimmerthal (1846) and his collection, which, as far as J. Vilbaste knew, had not been preserved (Vilbaste 1974). Subsequently, the questionable status of the species’ record in the Baltic States was emphasised in the Catalogue of the Auchenorrhyncha of Europe (Nast 1987) and in the Catalogue of the Auchenorrhyncha of Northern Europe (Söderman et al. 2009). At the same time, in the latter work, the authors stress that this species is difficult to confuse with any other Auchenorrhyncha in the region and suggest that B.A. Gimmerthal could hardly have made an identification error. In addition, this information was reported by Velce and Danka (1970) and was not questioned by Logvinenko (1975). The species has previously been recorded in Belarus (Borodin 2004).

In 2009, the species was found in the vicinity of Heggenes in Telemark, Norway (Ødegaard 2011). The specimens were collected between May 11 and June 2. It was erroneously reported from Lithuania by Anufriev and Egorov (2016); the paper cites works by Latvian specialists that record the species in Latvia.

The species is easily identifiable in the field, allowing additional information on its distribution to be obtained from iNaturalist data. The records available on gbif.org are also derived from iNaturalist. Thus, it can be stated that *A. clavicornis* is recorded for the first time with certainty in Lithuania. All records were made in the central, southern, and eastern parts of the country. Observations took place from April 19 to October 10.

### Stenocraninae Wagner, 1963

#### *Stenocranus* Fieber, 1866

***Stenocranus minutus* (Fabricius, 1787)**: Söderman et al. (2009); Valiūnas et al. (2017). Trak06; Trak08; Trak16 (Söderman and Rintala 2009); VilnD03; VilnD06 (Ivanauskas et al. 2014b). KauD04; KauD07; KauD17; Šal02; VilnC05.

*Stenocranus fuscovittatus (Stål, 1858) – AlytD01: 1♀, 23 Jun. 2022, sweep netting, A. Petrašiūnas leg.; AlytD02: 1♂ (MZVUHe00922), 24.08-04 Sept. 2018, Malaise trap, A. Petrašiūnas leg.; 1♂ (MZVUHe01349), 1♀, 24 Jun. 2020, sweep netting, A. Petrašiūnas leg.; KlaiD02: 4♀ (MZVUHe01283, MZVUHe01322, MZVUHe01323, MZVUHe01324), 25 Jun. 2020, sweep netting, A. Petrašiūnas leg.; K.R.02: 1♀, 30 May 2019, V. Tamutis leg.; Kel05: 1♂ (MZVUHe02327), Transition mire, 05 Aug. 2024, E. Sriubaitė leg.; 3♂ (MZVUHe02324, MZVUHe02325, MZVUHe02326), 3♀ (MZVUHe02321, MZVUHe02322, MZVUHe02329), 19 Aug. 2024, E. Sriubaitė leg.

**Remarks**. First registration for Lithuania. The species is very widely distributed in the Palearctic region (Dmitriev et al. 2022). Occurrence in Lithuania had been considered quite likely (Vilbaste 1974).

*Stenocranus major (Kirschbaum, 1868) – Kel03: 1♂ (MZVUHe02334), Alkaline fen, 19 Aug. 2024, E. Sriubaitė leg.; Kel05: 1♂ (MZVUHe02339), 1♀ (MZVUHe02323), Transition mire, 19 Aug. 2024, E. Sriubaitė leg.

**Remarks**. First registration for Lithuania. The species is very widely distributed in the western part of the Palearctic region (Dmitriev et al. 2022). According to published data, it has been recorded on *Phalaris arundinacea* and *Calamagrostis epigejos*; feeding on the second species requires confirmation (Holzinger et al. 2003; Nickel 2003).

### Kelisiinae Wagner, 1963

#### *Kelisia* Fieber, 1866

***Kelisia confusa* Linnavuori, 1957** (= *Kelisia nervosa* Vilbaste, 1972): Vilbaste (1960, 1972); Nickel (2003); Söderman et al. (2009); Malenovský and Lauterer (2010). Kal03; Mol02; PanD05; Šven03 (Vilbaste 1974). Kal02.

***Kelisia guttula* (Germar, 1818)**: Vilbaste (1974); Nast (1987); Söderman et al. (2009); Della Giustina (2019a); Karavin et al. (2023). Kel03; Kel05; Šiau06.

***Kelisia guttulifera* (Kirschbaum, 1868)**: Nast (1987); Nickel (2003); Söderman et al. (2009); Trivellone et al. (2015); Della Giustina (2019a). PanD01 (Vilbaste 1974).

***Kelisia monoceros* Ribaut, 1934**: Söderman et al. (2009).

***Kelisia pallidula* (Boheman, 1847)**: Vilbaste (1974); Nast (1987); Söderman et al. (2009); Della Giustina (2019a). PanC01 (Mensonienė 1979). AlytD02; AlytD05; Kel03; Kel05; KlaiD02; KlaiD07; Šiau06; Šven01.

***Kelisia praecox* Haupt, 1935**: Nast (1987); Söderman et al. (2009); Trivellone et al. (2015); Della Giustina (2019a). Kal03 (Vilbaste 1974). Kal02.

***Kelisia ribauti* Wagner, 1938**: Vilbaste (1974); Nast (1987); Demir (2008); Söderman et al. (2009); Della Giustina (2019a). Ign10.

***Kelisia sabulicola* Wagner, 1952**: Vilbaste (1974); Söderman et al. (2009); Karavin et al. (2023).

**Kelisia sima Ribaut, 1934 – Kel03: 1♀ (MZVUHe02222), Alkaline fen, 05 Aug. 2024, E. Sriubaitė leg.; 1♂ (MZVUHe02350), 19 Aug. 2024, E. Sriubaitė leg.; Kel05: 1♂ (MZVUHe02344), Transition mire, 05 Jul. 2024, E. Sriubaitė leg.; 2♂ (MZVUHe02205, MZVUHe02208), 19 Jul. 2024, E. Sriubaitė leg.; Šiau04: 1♀ (MZVUHe02215), Transition mire, 06 Aug. 2024, E. Sriubaitė leg.; Šiau06: 1♂ (MZVUHe02224), 3♀ (MZVUHe02209, MZVUHe02221, MZVUHe02214), 19 Jul. 2024, E. Sriubaitė leg.; 6♀ (MZVUHe02201, MZVUHe02218, MZVUHe02229, MZVUHe02232, MZVUHe02236, MZVUHe02238), 06 Aug. 2024, E. Sriubaitė leg.; 3♂ (MZVUHe02207, MZVUHe02217, MZVUHe02341), 19 Aug. 2024, E. Sriubaitė leg.

**Remarks**. The species is reported for the first time from the Baltic States. Morphologically, it is very similar to *Kelisia guttula* (Germar, 1818), and several publications have repeatedly emphasised the need to re-examine old collection material of this species from other parts of its range (Nickel 2003; Seljak 2016). Currently it has been recorded in Austria (Nast 1972, 1987; Holzinger et al. 2003; Nickel 2003; Malenovský and Lauterer 2010; Mühlethaler et al. 2018; Della Giustina 2019a), Czech Republic (Malenovský and Lauterer 2010), France (Nast 1972, 1987; Holzinger et al. 2003; Nickel 2003; Malenovský and Lauterer 2010), Germany (Nast 1972, 1987; Nickel and Remane 2002; Holzinger et al. 2003; Nickel 2003; Malenovský and Lauterer 2010; Mühlethaler et al. 2018; Della Giustina 2019a), Italy (Holzinger et al. 2003; Nickel 2003; Malenovský and Lauterer 2010; Della Giustina 2019a), Slovenia (Seljak 2016); Sweden (Holzinger et al. 2003; Nickel 2003; Söderman et al. 2009; Malenovský and Lauterer 2010; Della Giustina 2019a) and Switzerland (Mühlethaler et al. 2018; Della Giustina 2019a). *K. sima* has been reported to be a monophagous species on *Carex flava* (Nickel 2003; Della Giustina 2019a).

***Kelisia vittipennis* (Sahlberg, 1868)**: Vilbaste (1974); Nast (1987); Söderman et al. (2009). Kel05; Šiau02.

### Delphacinae Leach, 1815

**Delphacini Leach, 1815**

#### *Acanthodelphax* Le Quesne, 1964

***Acanthodelphax denticauda* (Boheman, 1847)**: Vilbaste (1974); Nast (1987); Söderman et al. (2009); Della Giustina (2019a). AlytD09; Kal02.

***Acanthodelphax spinosus* (Fieber, 1866)**: Vilbaste (1974); Nast (1987); Söderman et al. (2009); Della Giustina (2019a). AlytD01; AlytD09; B01; Drus01; Jona01; Jurb02; Mar01; Šilu01; Šven03; Tau02; Vilk01; VilnD07.

#### *Chloriona* Fieber, 1866

***Chloriona dorsata* Edwards, 1898**: Nast (1987); Söderman et al. (2009); Della Giustina (2019a). Ner04 (Vilbaste 1974).

*Chloriona glaucescens Fieber, 1866 – AlytD02: 1♂ (MZVUHe01609), 24 Jul. 2020, sweep netting, A. Petrašiūnas leg.; 1♂ (MZVUHe01852), 23 Jun. 2022, sweep netting, A. Petrašiūnas leg.

**Remarks**. The species has been widely recorded in many countries in Northern Europe, including Latvia, Estonia, and the Scandinavian countries (Söderman et al. 2009). It is also known from Poland (Gębicki et al. 2013) and Belarus (Borodin 2004). Like other species of the genus *Chloriona* Fieber, 1866, it is monophagous on *Phragmites australis*. In Central Europe, it prefers saline habitats and is less frequently found in freshwater sites (Holzinger et al. 2003; Nickel 2003).

***Chloriona smaragdula* (Stål, 1853)**: Vilbaste (1974); Nast (1987); Söderman et al. (2009). AlytD01; AlytD02; Anyk02; Ign02; Ign09; Šven28.

**Chloriona unicolor (Herrich-Schäffer, 1835) – Šiau07: 5♂ (MZVUHe02332, MZVUHe02333, MZVUHe02335, MZVUHe02336, MZVUHe02340), 05 Jul. 2024, E. Sriubaitė leg.

**Remarks**. A rare species. Like other members of the genus, *C. unicolor* is monophagous on *Phragmites australis*. It is distributed mainly in the southern part of the Palaearctic region (Della Giustina 2019a). Among the countries sharing a land border with Lithuania, it is known to occur only in southern and central Poland (Walczak et al. 2016). Records are also known from Finland (Asche 1982; Ding 2006; Kuznetsova et al. 2024) and Norway (Ding 2006). This is the first record from the Baltic States.

#### *Conomelus* Fieber, 1866

***Conomelus anceps* (Germar, 1821)**: Vilbaste (1974); Nast (1987); Söderman et al. (2009). Kel03; Kel05; KlaiD07; Šven01; Telš03; Var36; VilnD07.

#### *Criomorphus* Curtis, 1831

***Criomorphus albomarginatus* Curtis, 1833**: Vilbaste (1974); Nast (1987); Söderman et al. (2009). AlytD09; Drus01; Šak01.

*Criomorphus moestus (Boheman, 1847) – AlytD01: 1♂ (MZVUHe01818), 24 Jun. 2020, sweep netting, A. Petrašiūnas leg.

**Remarks**. It is known from neighbouring countries, including Latvia (Borodin and Borodina 2021) and Belarus (Logvinenko 1984; Nast 1987; Borodin 2015a), and has also been recorded in Finland and Sweden (Söderman et al. 2009). Its host plant is reported to be *Calamagrostis canescens* (Holzinger et al. 2003; Nickel 2003).

#### *Delphacinus* Fieber, 1866

***Delphacinus mesomela* (Boheman, 1850)**: Vilbaste (1974); Nast (1987); Söderman et al. (2009). Anyk01.

#### *Delphax* Fabricius, 1798

***Delphax crassicornis* (Panzer, 1796)**: Vilbaste (1974); Nast (1987); Söderman et al. (2009). AlytD04; Kaiš06; Kup05; Šiau06; Šilu14; Trak17; Var28.

***Delphax pulchellus* (Curtis, 1833)**: Nast (1987); Söderman et al. (2009). AlytD09 (Vilbaste 1974). AlytD01; AlytD02; Ign02; KlaiD07; Šilu10.

#### *Dicranotropis* Fieber, 1866

***Dicranotropis hamata* (Boheman, 1847)**: Vilbaste (1974); Nast (1987); Söderman et al. (2009). PanC01 (Mensonienė 1979). Šilu01; Šilu02.

#### *Euconomelus* Haupt, 1929

***Euconomelus lepidus* (Boheman, 1847)**: Vilbaste (1974); Nast (1987); Söderman et al. (2009). AlytD05; Anyk02; KlaiD07; Laz06; Telš03; Var36.

#### *Euides* Fieber, 1866

*Euides speciosa (Boheman, 1845) (= *Euides basilinea* (Germar, 1821)) **–** AlytD02: 1♂ (MZVUHe01874), 23 Jun. 2022, sweep netting, A. Petrašiūnas leg.; Jona01: 1♂ (MZVUHe01271), 15–26 Jun. 2022, Malaise trap, A. Petrašiūnas leg.

**Remarks**. The host plant is *Phragmites australis* (Holzinger et al. 2003; Nickel 2003; Della Giustina 2019a; Gjonov 2022). A European species, it is the only representative of the genus *Euides* Fieber, 1866 occurring in Northern Europe (Della Giustina 2019a). In particular, it has been reported as *E. basilinea* from Latvia (Borodin and Borodina 2021), Poland (Gębicki et al. 2013), and Belarus (Borodin 2004).

#### *Eurybregma* Scott, 1875

*Eurybregma nigrolineata Scott, 1875 – Šal02: 1♂, 08 Jun. 2019, V. Tamutis leg.

**Remarks**. In Northern Europe, the species had previously been reported only from Sweden (Söderman et al. 2009). In 2021, it was found in eastern Latvia (Borodin 2023). Both brachypterous and macropterous forms were recorded. It is a Euro-Siberian species (Gębicki et al. 2013), whose range expansion in Europe towards the northwest has been noted since the mid-20^th^ century. However, the species has been known from England since the 19^th^ century (including brachypterous forms) (Nickel 2003). This likely explains why J. Vilbaste did not record the species during mid-20^th^-century intensive studies on leafhoppers in Estonia, Latvia, and Lithuania. In addition to the currently known Latvian records, findings from western Estonia were made between 2016 and 2018. These specimens are stored in the collection of the Natural History Museum of the University of Tartu (TUZ).

#### *Eurysa* Fieber, 1866

***Eurysula lurida* (Fieber, 1866)**: Nast (1987); Söderman et al. (2009); Gjonov (2022). Drus01; Maž02; Šven14 (Vilbaste 1974). Kal02.

#### *Gravesteiniella* Wagner, 1963

***Gravesteiniella boldi* (Scott, 1870)**: Vilbaste (1974); Nast (1987); Söderman et al. (2009); Ramsay (2016). AlytD05.

#### *Hyledelphax* Vilbaste, 1968

***Hyledelphax elegantula* (Boheman, 1847)**: Vilbaste (1974); Nast (1987); Söderman et al. (2009).

#### *Javesella* Fennah, 1963

***Javesella discolor* (Boheman, 1847)**: Vilbaste (1974); Nast (1987); Söderman et al. (2009).

***Javesella dubia* (Kirschbaum, 1868)**: Vilbaste (1974); Nast (1987); Söderman et al. (2009). AlytD02; AlytD05; Drus01; KlaiD07; Telš02.

***Javesella forcipate* (Boheman, 1847)**: Vilbaste (1974); Nast (1987); Söderman et al. (2009). K.R.01; Ras02; Telš02.

***Javesella obscurella* (Boheman, 1847)**: Vilbaste (1974); Nast (1987); Söderman et al. (2009). B01; Drus01; KlaiD07; Laz06.

***Javesella pellucida* (Fabricius, 1794)**: Vilbaste (1974); Nast (1987); Demir (2008); Söderman et al. (2009). PanC01 (Mensonienė 1979); KauD14; VilnD06 (Ivanauskas et al. 2014b). AlytD01; AlytD02; Anyk05; Anyk06; B01; Ign02; Ign09; Jurb06; Kel03; Kel05; KlaiD07; Kup03; Kup05; Ner02; Ner05; Pag01; PanD04; Rok06; Šiau02; Šiau06; Šilu04; Šilu10; Šilu12; Šven01; Šven06; Var28.

***Javesella salina* (Haupt, 1924)**: Nast (1987); Demir (2008); Söderman et al. (2009); Della Giustina (2019b). AlytD06 (Vilbaste 1974). AlytD05.

***Javesella tapina* (Fieber, 1866)** (= *J. stali* (Metcalf, 1943)): Vilbaste (1974); Nast (1987); Söderman et al. (2009). B01; Drus01; Laz06; Mar01.

#### *Kosswigianella* Wagner, 1963

***Kosswigianella exigua* (Boheman, 1847)**: Vilbaste (1974); Nast (1987); Söderman et al. (2009). B01; Trak18; Var36; Var37.

#### *Laodelphax* Fennah, 1963

***Laodelphax striatellus* (Fallén, 1826)**: Nast (1987); Demir (2008); Söderman et al. (2009). Var37 (Vilbaste 1974); PanC01 (Mensonienė 1979). AlytD01; AlytD02; Ign02; Kel03; Kel05; Kup03; Šilu10; Šilu11; Šilu12.

#### *Megadelphax* Wagner, 1963

*Megadelphax sordidula (Stål, 1853) – Pag01: 1♂ (MZVUHe01638), 12 Aug. 2022, yellow pan traps, J. Skuja leg.; 2♂ (MZVUHe00683, MZVUHe00685), 04 Sept. 2022, yellow pan traps, G. Skujienė leg.

**Remarks**. A widespread species occurring throughout most of Europe and in North Africa. The eastern limit of its range extends to Mongolia (Vilbaste 1971; Nast 1972, 1987; Logvinenko 1975). In Northern Europe, it is known from Sweden, Finland, Estonia, Latvia, and Karelia (Söderman et al. 2009). It has also been recorded in Belarus (Borodin 2004) and Poland (Gębicki et al. 2013). Its host plant is reported to be *Arrhenatherum elatius* (Nickel 2003; Gębicki et al. 2013).

#### *Megamelus* Fieber, 1866

***Megamelus notula* (Germar, 1830)**: Vilbaste (1974); Nast (1987); Söderman et al. (2009). Ign09; Šilu05; Šilu10; Šilu12; Šven01; Šven16.

#### *Muellerianella* Wagner, 1963

***Muellerianella brevipennis* (Boheman, 1847)**: Vilbaste (1974); Nast (1987); Söderman et al. (2009). K.R.01; Mar01; Šilu12; Šven03.

***Muellerianella extrusa* (Scott, 1871)**: Nast (1987). Šiau05; Šven01.

***Muellerianella fairmairii* (Perris, 1857)**: Vilbaste (1974); Nast (1987); Söderman et al. (2009).

#### *Muirodelphax* Wagner, 1963

***Muirodelphax aubei* (Perris, 1857)**: Vilbaste (1974); Nast (1987); Demir (2008); Söderman et al. (2009). Ign06; Var36; VilnD07.

#### *Nothodelphax* Fennah, 1963

***Nothodelphax distinctus* (Flor, 1861)** (= *Tyrphodelphax distinctus* (Flor, 1861)): Vilbaste (1974); Nast (1987); Söderman et al. (2009). AlytD09; Šven12.

#### *Oncodelphax* Wagner, 1963

***Oncodelphax pullulan* (Boheman, 1852)**: Vilbaste (1974); Nast (1987); Söderman et al. (2009); Gjonov (2022). Anyk01.

#### *Paradelphacodes* Wagner, 1963

***Paradelphacodes paludosa* (Flor, 1861)**: Nast (1987); Söderman et al. (2009). Šila01 (Vilbaste 1974). AlytD02; Šiau06.

#### *Paraliburnia* Jensen-Haarup, 1917

***Paraliburnia adela* (Flor, 1861)**: Nast (1987); Söderman et al. (2009); Trivellone (2010); Mitjaev (2015). PanD04 (Vilbaste 1974). Šilu05.

**Paraliburnia clypealis (Sahlberg, 1871) – AlytD02: 1♂ (MZVUHe00048), 14–23 Jun. 2022, Malaise trap, A. Petrašiūnas leg.

**Remarks**. Known from the Czech Republic (Nast 1972, 1987; Nickel 2003), England (Nast 1972, 1987; Mitjaev 2015; Foster 2019), Finland (Nast 1972, 1987; Söderman et al. 2009; Albrecht et al. 2015; Mitjaev 2015; Della Giustina 2019b), France (Della Giustina 2019b), Germany (Nast 1972, 1987; Nickel and Remane 2002; Nickel 2003; Mitjaev 2015; Mühlethaler et al. 2018; Della Giustina 2019b), Ireland (Wilson et al. 2015; Della Giustina 2019b), Kazakhstan (Mitjaev 2002, 2015), Luxembourg (Den Bieman et al. 2011; Della Giustina 2019b), the Netherlands (Nast 1987; Nickel 2003; Den Bieman et al. 2011; Della Giustina 2019b), Poland (Gębicki et al. 2013), the European part of Russia (north – Nast 1972, 1987; Mitjaev 2015; centre – Anufriev and Smirnova 2009), and Sweden (Nast 1972, 1987; Söderman et al. 2009; Mitjaev 2015). This is the first record from the Baltic States.

#### *Ribautodelphax* Wagner, 1963

***Ribautodelphax albostriata* (Fieber, 1866)**: Vilbaste (1974); Nast (1987); Söderman et al. (2009). AlytD09; Drus01; Ign04; Vilk01.

***Ribautodelphax collina* (Boheman, 1847)**: Vilbaste (1974); Nast (1987); Söderman et al. (2009). B01.

***Ribautodelphax pallens* (Stål, 1854)**: Vilbaste (1974); Nast (1987); Söderman et al. (2009). AlytD09; Šilu02.

#### *Stiroma* Fieber, 1866

***Stiroma affinis* Fieber, 1866**: Vilbaste (1974); Nast (1987); Söderman et al. (2009). AlytD08; PanC01; Rok03.

***Stiroma bicarinatum* (Herrich-Schäffer, 1835)**: Vilbaste (1974); Nast (1987); Söderman et al. (2009). Šven29.

#### *Struebingianella* Wagner, 1963

***Struebingianella lugubrina* (Boheman, 1847)**: Vilbaste (1974); Nast (1987); Söderman et al. (2009). Anyk01; K.R.01; K.R.02; PanD05.

***Struebingianella paryphasma* (Flor, 1861)** (= *Florodelphax paryphasma* (Flor, 1861)): Nast (1987); Preisler and Lauterer (2003); Söderman et al. (2009); Gjonov (2022). B01; Zar04 (Vilbaste 1974). AlytD02.

#### *Unkanodes* Fennah, 1956

***Unkanodes excisa* (Melichar, 1898)**: Nast (1987); Söderman et al. (2009). Drus01; PalC01 (Vilbaste 1974). Drus05.

#### *Xanthodelphax* Wagner, 1963

***Xanthodelphax flaveola* (Flor, 1861)**: Vilbaste (1974); Nast (1987); Söderman et al. (2009).

***Xanthodelphax straminea* (Stål, 1858)**: Vilbaste (1974); Nast (1987); Söderman et al. (2009). KauD14 (Ivanauskas et al. 2014b). KlaiD07; Šilu02.

### Achilidae Stål, 1866

**Achilinae Stål, 1866**

#### *Cixidia* Fieber, 1866

***Cixidia confinis* (Zetterstedt, 1837)**: Söderman et al. (2009). Var01 (Söderman and Dapkus 2009). Tau01.

### Caliscelidae Amyot & Audinet-Serville, 1843

**Ommatidiotinae Fieber, 1875**

#### *Ommatidiotus* Spinola, 1839

***Ommatidiotus dissimilis* (Fallén, 1806)**: Vilbaste (1974); Söderman et al. (2009). Kel03; Kel05; KlaiD02; Ner04; Šilu07; Šilu08; Šven01; Šven06; Šven12; Šven18; Šven25; Šven27; Šven29.

### Tettigometridae Germar, 1821

**Tettigometrinae Germar, 1821**

#### *Tettigometra* Latreille, 1804

***Tettigometra atra* Hagenbach, 1825** (= *T. atrata* Fieber, 1872): Nast (1987); Söderman et al. (2009). VilnD07 (Vilbaste 1974). KauC06.

### Cicadomorpha Evans, 1946

**Cicadidae Batsch, 1789**

**Cicadettinae Buckton, 1890**

**Cicadettini Buckton, 1890**

#### *Cicadetta* Kolenati, 1857

***Cicadetta montana* (Scopoli, 1772) s.str**.: KauC04; KauD15; KauD21 (Švitra 2007). KauC03; KauC05; KauC08; KauD20; KauD22.

### Cercopoidea Leach, 1815

**Aphrophoridae Amyot & Audinet-Serville, 1843**

**Aphrophorinae Amyot & Audinet-Serville, 1843**

**Aphrophorini Amyot & Audinet-Serville, 1843**

#### *Aphrophora* Germar, 1821

***Aphrophora alni* (Fallén, 1805)**: Vilbaste (1974); Nast (1987); Söderman et al. (2009); Ivanauskas et al. (2014a). PanC01 (Mensonienė 1979); KauD14; VilnD03; VilnD06 (Ivanauskas et al. 2014b). AlytD01; AlytD09; Drus01; Ign02; Ign09; Jurb01; K.R.03; Kaiš01; Kaiš02; KauD15; Kel03; Kel05; Ner07; PanD03; Prie01; Rok02; Rok04; Rok07; Rok08; Šak13; Šiau02; Šiau06; Šilu04; Šilu06; Šilu10; Šilu11; Šilu14; Širv01; Šven01; Šven15; Šven19; Šven27; Trak12; Ute04; Var01; Var28.

**Aphrophora corticea Germar, 1821 – Šak01: 1♂, 06 Jul. 2013, leg R. Ferenca (stored in KTIZM).

iNaturalist record: KlaiD01, 06 Sept. 2020 (iN65898025).

**Remarks**. It occurs in Western and Central Europe, in southern Scandinavia, and has been recorded in the Balkans, the Altai Mountains, Anatolia, and Siberia (Holzinger et al. 2003). In particular, it is known from Norway and Sweden (Söderman et al. 2009), Poland (Gębicki et al. 2013), and Belarus (Borodin 2015a). This is the first record from the Baltic States.

***Aphrophora major* Uhler, 1896**: Söderman et al. (2009). Var01 (Söderman and Dapkus 2009). AlytD01; AlytD02; Ign02.

***Aphrophora salicina* (Goeze, 1778)** (= *A. costalis* Matsumura, 1903, *A. pectoralis* Matsumura, 1903): Vilbaste (1974); Nast (1987); Söderman et al. (2009). Ign09; Jurb04; KauC07; Kel05; KlaiD02; KlaiD05; Mol04; Ner07; PanD01; Rok04; Šiau06.

#### *Peuceptyelus* Sahlberg, 1871

***Peuceptyelus coriaceus* (Fallén, 1826)**: Nast (1987); Holzinger et al. (2003); Söderman et al. (2009). PanC01 (Mensonienė 1979); B01 (Vilbaste 1974). Ign01; Ute02.

### Cloviini Schmidt, 1920

#### *Lepyronia* Amyot & Audinet-Serville, 1843

***Lepyronia coleoptrata* (Linnaeus, 1758)**: Vilbaste (1974); Nast (1987); Söderman et al. (2009); Ivanauskas et al. (2014a). KauD14 (Ivanauskas et al. 2014b). Akm01; AlytD01; AlytD02; Ign02; Ign03; Ign09; Jurb04; Jurb05; KauD15; Kel03; Kel05; KlaiD02; KlaiD07; Ras03; Rok07; Šak04; Šiau02; Šiau06; Šilu03; Šilu04; Šilu05; Šilu10; Šilu11; Šilu12; Šven30; Tau05; Ute02; Ute04; Var23.

### Philaenini Metcalf, 1955

#### *Neophilaenus* Haupt, 1935

***Neophilaenus campestris* (Fallén, 1805)**: Vilbaste (1974); Nast (1987); Anufriev and Kirillova (1998); Demir (2008); Söderman et al. (2009). AlytD05; Var37.

***Neophilaenus exclamationis* (Thunberg, 1784)**: Vilbaste (1974); Nast (1987); Söderman et al. (2009). Anyk01.

***Neophilaenus lineatus* (Linnaeus, 1758)** (= *N. pallidus* (Haupt, 1917)): Vilbaste (1974); Nast (1987); Söderman et al. (2009). AlytD01; AlytD02; AlytD09; Ign02; Ign09; Jurb01; Kel03; Kel04; KlaiD02; KlaiD07; Ner01; Ner04; Ner05; Rok02; Rok07; Šiau02; Šiau06; Šilu04; Šilu05; Šilu06; Šilu12; Šilu13; Šilu14; Šven01; Šven06; Šven11; Šven12; Šven15; Šven16; Šven25; Šven27; Var01.

***Neophilaenus minor* (Kirschbaum, 1868)**: Vilbaste (1974); Nast (1987); Nickel (2003); Demir (2008); Söderman et al. (2009). Anyk01; Laz06; Šven24; Var17; Var37.

#### *Philaenus* Stål, 1864

***Philaenus spumarius* (Linnaeus, 1758)**: Vilbaste (1974); Nast (1987); Demir (2008); Söderman et al. (2009); Ivanauskas et al. (2014a). PanC01 (Mensonienė 1979); KauD14; VilnD03; VilnD06 (Ivanauskas et al. 2014b). AlytD01; AlytD02; AlytD09; Bir01; K.R.03; Kaiš01; Kaiš02; Kaiš03; Kal02; KauC08; KauD04; KlaiD06; KlaiD07; Kup03; Ner02; Ner07; Ner09; PalM02; Rok02; Rok04; Rok05; Rok06; Rok07; Rok08; Šak11; Šiau06; Šilu03; Šilu04; Šilu05; Šilu06; Šilu10; Šilu11; Šilu12; Šilu13; Šilu14; Šven28; Ute04; Var01; Var28; Var41; Vilk02; Zar02.

### Membracoidea Rafinesque, 1815

**Membracidae Rafinesque, 1815**

**Centrotinae Amyot & Audinet-Serville, 1843**

**Centrotini Amyot & Audinet-Serville, 1843**

#### *Centrotus* Fabricius, 1803

***Centrotus cornutus* (Linnaeus, 1758)**: Vilbaste (1974); Nast (1987); Demir (2008); Söderman et al. (2009); Petrašiūnas (2021). AlytD09; Bir02; K.R.04; Kaiš04; KauC07; KauC09; KauD04; KauD05; KauD08; KauD09; KauD10; KauD11; KauD15; KauD16; KauD24; Laz06; Šak13; Šal01; Šal02; Šiau05; Šiau07; Šven28.

### Gargarini Distant, 1908

#### *Gargara* Amyot & Audinet-Serville, 1843

***Gargara genistae* (Fabricius, 1775)**: Var15; Var30 (Petrašiūnas 2021); Var02 (Petrašiūnas 2021, 2023). AlytC02; AlytC03; AlytD03; Anyk04; Drus02; Drus03; Drus04; El03; Kaiš05; Kel01; Kel02; KlaiC02; KlaiC03; KlaiD03; Kre01; Laz03; Laz04; Maž01; Ner03; PalM01; Plu01; Plu02; Prie06; Prie07; Rad02; Rad03; Šak02; Šak17; Širv02; Šven02; Šven05; Šven09; Šven21; Šven22; Šven31; Šven32; Šven33; Telš01; Telš04; Trak09; Trak11; Ute06; Ute07; Var02; Var03; Var05; Var07; Var09; Var10; Var11; Var14; Var16; Var18; Var19; Var21; Var22; Var24; Var25; Var27; Var30; Var31; Var32; Var33; Var35; Var38; Var39; Var40; VilnC06 (Petrašiūnas 2023). Šven01; Var13; Var16; Var28.

### Smiliinae Stål, 1866

**Ceresini Goding, 1892**

#### *Stictocephala* Stål, 1869

***Stictocephala bisonia* Kopp & Yonke, 1977**: Bogoutdinov et al. (2024). Var29 (Petrašiūnas 2021). Var04.

### Cicadellidae Latreille, 1825

**Ulopinae Le Peletier & Audinet-Serville, 1828**

#### *Ulopa* Fallén, 1814

***Ulopa reticulata* (Fabricius, 1794)**: Vilbaste (1974); Nast (1987); Szwedo and Gębicki (2001); Söderman et al. (2009). Bir01; KauD02; KauD04; Ner05; Šilu07; Šilu08; Šven08; Šven15; Šven17; Šven20; Šven25; Šven27.

#### *Utecha* Emeljanov, 1996

***Utecha trivia* (Germar, 1821)**: Söderman et al. (2009); Söderman and Rintala (2009). Ign08; Kup01; Trak12; Trak13.

### Ledrinae Fairmaire, 1855

#### *Ledra* Fabricius, 1803

***Ledra aurita* (Linnaeus, 1758)**: KauC01 (Gimmerthal 1846); VilnC13 (Ivanauskas et al. 2014b). KauD19; Prie02; Šak14.

### Megophthalminae Kirkaldy, 1906 [1859]

**Agalliini Kirkaldy, 1901**

#### *Agallia* Curtis, 1833

***Agallia brachyptera* (Boheman, 1847)**: Vilbaste (1974); Nast (1987); Söderman et al. (2009). PanC01 (Mensonienė 1979). Jurb05; Mol01; Pag01; Rok06; Šilu10; Šilu11; Šilu12; Šilu14; Trak12; Trak13; Trak14; Trak17; Var37.

#### *Anaceratagallia* Zakhvatkin, 1946

***Anaceratagallia ribauti* (Ossiannilsson, 1938)** (= *A. lithuanica* Vilbaste, 1974): Nast (1982, 1987); Söderman et al. (2009); Ivanauskas et al. (2014a); VilnD07 (Vilbaste 1974); KauD14 (Ivanauskas et al. 2014b). AlytD05; Kup02; PanD01; Var37.

***Anaceratagallia venosa* (Fourcroy, 1785)**: Vilbaste (1974); Nast (1987); Söderman et al. (2009). Jurb02; Laz06; Var37.

### Megophthalmini Kirkaldy, 1906 [1859]

***Megophthalmus scanicus* (Fallén, 1806)**: Vilbaste (1974); Nast (1987); Söderman et al. (2009). AlytD01; AlytD04; AlytD05; Bir03; KauD18; Kup01; Kup02; Ras01; Šila01; Šilu01; Šilu02; Šilu05; Šilu10; Šilu12; Šven19; Šven29; Telš03; VilnC01.

### Eurymelinae Amyot & Audinet-Serville, 1843

**Macropsini Evans, 1936**

#### *Hephathus* Ribaut, 1952

***Hephathus achilleae* Mitjaev, 1967**: Söderman et al. (2009).

***Hephathus nanus* (Herrich-Schäffer, 1835)**: Vilbaste (1974); Nast (1987); Trivellone et al. (2015). Laz06; Trak15; Trak18; Ute03; Var17; Var36.

#### *Macropsidius* Ribaut, 1952

***Macropsidius sahlbergii* (Flor, 1861)**: Nast (1987); Söderman et al. (2009). Šven24 (Vilbaste 1974). Laz06.

#### *Macropsis* Lewis, 1834

***Macropsis cerea* (Germar, 1837)**: Vilbaste (1974); Nast (1987); Söderman et al. (2009); Dai et al. (2018). AlytD04; Anyk01; Bir01; Jurb02; PanD05; Šila01; Ute01; VilnD07; VilnD11.

***Macropsis fuscinervis* (Boheman, 1845)**: Vilbaste (1974); Nast (1987); Söderman et al. (2009); Dai et al. (2018). Ign09; K.R.01; Laz06.

***Macropsis fuscula* (Zetterstedt, 1828)**: Vilbaste (1974); Söderman et al. (2009). Ign02; Jurb02; KauD01; Laz06; PanD01; Šven03; Šven14; Var12; Zar01; Zar04.

***Macropsis glandacea* (Fieber, 1868)**: Söderman et al. (2009).

***Macropsis impura* (Boheman, 1847)**: Vilbaste (1974); Nast (1987); Söderman et al. (2009). Bir04; PanC01; Ute01; VilnD07.

***Macropsis infuscata* (Sahlberg, 1871)**: Vilbaste (1974); Nast (1987); Söderman et al. (2009). Bir04; El02; Ign04; Šven24; Telš05.

***Macropsis marginata* (Herrich-Schäffer, 1835)**: Vilbaste (1974); Nast (1987); Söderman et al. (2009). Bir01; Kėd01; Mar01; PanD01; Rad04; Ras04; Šila01; VilnD07.

***Macropsis mendax* (Fieber, 1868)**: Nast (1987). AlytC01 (Vilbaste 1974).

***Macropsis ocellata* Provancher, 1872** (= *M. albae* Wagner, 1950): Vilbaste (1974); Nast (1987); Demir (2008); Söderman et al. (2009). Jurb02; VilnD07.

***Macropsis prasina* (Boheman, 1852)**: Vilbaste (1974); Nast (1987); Söderman et al. (2009). Šilu02; Tau02.

***Macropsis vicina* (Horváth, 1897)**: Söderman et al. (2009). Trak04; Trak07; Var06 (Söderman and Rintala 2009).

***Macropsis viridinervis* Wagner, 1950**: Söderman et al. (2009). Mar01; Var08; VilnD07 (Vilbaste 1974). Šiau02.

#### *Oncopsis* Burmeister, 1838

***Oncopsis alni* (Schrank, 1801)**: Vilbaste (1974); Nast (1987); Söderman et al. (2009); Dai et al. (2018). Ner04.

***Oncopsis appendiculata* Wagner, 1944**: Nast (1987); Söderman et al. (2009). Bir04 (Vilbaste 1974).

***Oncopsis carpini* (Sahlberg, 1871)**: Nast (1987); Söderman et al. (2009). AlytD04; KlaiD04; PanD01 (Vilbaste 1974).

***Oncopsis flavicollis* (Linnaeus, 1761)**: Vilbaste (1974); Nast (1987); Söderman et al. (2009). Anyk02; Ner07; Ner08; Šak09; Šilu02; Šven25; Šven29.

***Oncopsis subangulata* (Sahlberg, 1871)**: Nast (1987); Söderman et al. (2009). Šilu02 (Vilbaste 1974).

***Oncopsis tristis* (Zetterstedt, 1837)**: Vilbaste (1974); Nast (1987); Demir (2008); Söderman et al. (2009).

#### *Pediopsis* Burmeister, 1838

*Pediopsis tiliae (Germar, 1831) – Kre02: 1♀, 28 Aug. 1996, R. Ferenca leg.

**Remarks**. Feeds on species of the genus *Tilia* (Nickel 2003). A widespread species (Dmitriev et al. 2022). In Northern Europe, it is known from Scandinavia, Denmark, and Latvia (Söderman et al. 2009). It has also been recorded in Poland (Gębicki et al. 2013) and Belarus (Borodin 2004). Its occurrence in Lithuania was expected; however, the species had not been recorded for more than 30 years, despite its trophic association with host plants widespread in Lithuania and its ease of identification in the field.

### Idiocerini Baker, 1915

#### *Acericerus* Dlabola, 1974

***Acericerus ribauti* Nickel & Remane, 2002**: Söderman et al. (2009); Trak01 (Söderman and Rintala 2009). KauC01.

#### *Idiocerus* Lewis, 1834

***Idiocerus lituratus* (Fallén, 1806)**: Vilbaste (1974); Nast (1987); Söderman et al. (2009). AlytD04; AlytD05; AlytD09; Jona01; Kel05; Ner01; Šiau02; Šven03.

***Idiocerus similis* Kirschbaum, 1868**: Nast (1987); Söderman et al. (2009). Jurb02; PanD04; VilnD07 (Vilbaste 1974).

***Idiocerus stigmaticalis* Lewis, 1834**: Vilbaste (1974); Nast (1987); Söderman et al. (2009). Jurb02; Jurb04; Telš02; VilnD07.

#### *Metidiocerus* Ossiannilsson, 1981

***Metidiocerus elegans* (Flor, 1861)** (= *Idiocerus elegans* Flor, 1861): Vilbaste (1974); Nast (1987); Söderman et al. (2009). Trak03; Ute01.

#### *Populicerus* Dlabola, 1974

***Populicerus albicans* (Kirschbaum, 1868)**: Söderman et al. (2009). Trak04; Trak07 (Söderman and Rintala 2009). Tau05.

***Populicerus confusus* (Flor, 1861)** (= *Idiocerus confusus* Flor, 1861): Vilbaste (1974); Nast (1987); Söderman et al. (2009). Šilu04; Šven24; Ute01; Ute05.

***Populicerus nitidissimus* (Herrich-Schäffer, 1835)**: Söderman et al. (2009). Trak07 (Söderman and Rintala 2009).

***Populicerus populi* (Linnaeus, 1761)** (= *Idiocerus populi* (Linnaeus, 1761)): Vilbaste (1974); Nast (1987); Söderman et al. (2009). El02; KauD04; PanD01; Šak13; Šven08; Šven10; Var28.

#### *Rhytidodus* Fieber, 1872

***Rhytidodus decimaquartus* (Schrank, 1776)**: Vilbaste (1974); Nast (1987); Anufriev and Kirillova (1998); Söderman et al. (2009). Šven24.

#### *Stenidiocerus* Ossiannilsson, 1981

***Stenidiocerus poecilus* (Herrich-Schäffer, 1835)** (= *Idiocerus poecilus* (Herrich-Schäffer, 1835)): Nast (1987); Anufriev and Kirillova (1998); Söderman et al. (2009). PalM04 (Vilbaste 1974); Var01 (Söderman and Dapkus 2009).

#### *Tremulicerus* Dlabola, 1974

***Tremulicerus distinguendus* (Kirschbaum, 1868)**: Söderman et al. (2009). Trak01; Var06 (Söderman and Rintala 2009).

***Tremulicerus fulgidus* (Fabricius, 1775)** (= *Idiocerus fulgidus* (Fabricius, 1775)): Nast (1987); Söderman et al. (2009). Šven24 (Vilbaste 1974).

***Tremulicerus tremulae* (Estlund, 1796)** (= *Idiocerus tremulae* (Estlund, 1796)): Nast (1987); Anufriev and Kirillova (1998); Söderman et al. (2009). AlytC01; Jurb02; K.R.01 (Vilbaste 1974). Kaiš06.

#### *Viridicerus* Dlabola, 1974

***Viridicerus ustulatus* (Mulsant & Rey, 1855)**: Söderman et al. (2009). Trak02; Trak04; Trak07; Var06 (Söderman and Rintala 2009).

### Iassinae Walker, 1869

**Batracomorphini Krishnankutty, Dietrich, Dai & Siddappaji, 2016**

#### *Batracomorphus* Lewis, 1834

***Batracomorphus allionii* (Turton, 1802)**: Nast (1987); Söderman et al. (2009); Hu et al. (2021). Mar01; PanD01; Trak03 (Vilbaste 1974). Kaiš06; Mar01; PanD01; Trak03.

***Batracomorphus irroratus* Lewis, 1834**: Nast (1987); Demir (2008); Söderman et al. (2009); Hu et al. (2021); Abdollahi et al. (2015). Ign06; Laz06; Var36 (Vilbaste 1974).

### Iassini Walker, 1869

#### *Iassus* Fabricius, 1803

***Iassus lanio* (Linnaeus, 1761)**: Vilbaste (1974); Nast (1987); Söderman et al. (2009). AlytD04; Bir04; El02; Laz05; PanD05; Šak08; Šven03; Telš02; Vilk01.

### Aphrodinae Haupt, 1927 [1859]

**Aphrodini Haupt, 1927 [1859]**

#### *Anoscopus* Kirschbaum, 1858

***Anoscopus albiger* (Germar, 1821)**: PanC01 (Mensonienė 1979).

***Anoscopus flavostriatus* (Donovan, 1799)** (= *Aphrodes flavostriatus* (Donovan, 1799)): Vilbaste (1974); Söderman et al. (2009). PanC01 (Mensonienė 1979). Kup02; Pag01; PanD04; Šilu11; Šilu12; Telš03; Trak12; Trak13; Trak14; Trak17.

***Anoscopus histrionicus* (Fabricius, 1794)** (= *Aphrodes histrionicus* (Fabricius, 1794)): Söderman et al. (2009). AlytC01; Laz06; Šven24 (Vilbaste 1974).

***Anoscopus serratulae* (Fabricius, 1775)** (= *Aphrodes serratulae* (Fabricius, 1775)): Nast (1987); Söderman et al. (2009). Anyk01; Ign04; Ute01 (Vilbaste 1974). Kup01; Šilu12.

#### *Aphrodes* Curtis, 1831

*Aphrodes bicincta* (Schrank, 1776) (= *A. ochromelas* (Gmelin, 1789)): Vilbaste (1974); Nast (1987); Demir (2008); Söderman et al. (2009); Abdollahi et al. (2015). PanC01 (Mensonienė 1979). AlytD05; AlytD09; Kup02; Laz06; Rie01; Šilu05; Šilu11; Šilu12; Šven03; Šven11; Šven24; Trak12; Trak14; Trak15; Trak17; Ute03; Var36.

*Aphrodes diminuta* Ribaut, 1952: Söderman et al. (2009). Kup02.

*Aphrodes makarovi* Zakhvatkin, 1948: Nast (1987); Demir (2008); Söderman et al. (2009). Anyk01; Jurb02; Laz06 (Vilbaste 1974). Trak14.

#### *Planaphrodes* Hamilton, 1975

***Planaphrodes bifasciata* (Linnaeus, 1758)** (= *Aphrodes bifasciata* (Linnaeus, 1758)): Vilbaste (1974); Nast (1987); Söderman et al. (2009); Liang et al. (2023). PanC01 (Mensonienė 1979). Anyk01; Ign06; Laz06; Ras01; Šiau03; Šilu01; Šilu02; Telš03; Trak18.

***Planaphrodes laeva* (Rey, 1891)** (= *Aphrodes trifasciatus* (G., 1785), *P. trifasciatus* (Fourcroy, 1785)): Demir (2008); Söderman et al. (2009); Liang et al. (2023). Ign06; Laz06.

***Planaphrodes nigrita* (Kirschbaum, 1868)** (= *Aphrodes nigritus* (Kirschbaum, 1868)): Söderman et al. (2009). AlytC01; Bir01; Laz02; PanC01; Vilk01 (Vilbaste 1974).

#### *Stroggylocephalus* Flor, 1861

***Stroggylocephalus agrestis* (Fallén, 1806)**: Vilbaste (1974); Nast (1987); Söderman et al. (2009); Trivellone (2010). AlytD02; Ign10; Kel05; KlaiD07; Šiau02; Šilu10; Šilu11; Šilu12; Telš03; Trak03; Ute01.

***Stroggylocephalus livens* (Zetterstedt, 1837)**: Nast (1987); Söderman et al. (2009). AlytD09 (Vilbaste 1974). KlaiD02.

### Evacanthinae Crumb, 1911

**Evacanthini Crumb, 1911**

#### *Evacanthus* Le Peletier & Audinet-Serville, 1828

***Evacanthus acuminatus* (Fabricius, 1794)**: Vilbaste (1974); Nast (1987); Demir (2008); Söderman et al. (2009). Bir01; PanD05; Šven24.

***Evacanthus interruptus* (Linnaeus, 1758)**: Vilbaste (1974); Nast (1987); Söderman et al. (2009). Bir01; KauD13; Kup01; Kup03; Mar01; Šilu14; Šven24; Telš03; Trak12; Var28.

### Cicadellinae Latreille, 1825

**Cicadellini Latreille, 1825**

#### *Cicadella* Latreille, 1817

***Cicadella viridis* (Linnaeus, 1758)**: Vilbaste (1974); Nast (1987); Söderman et al. (2009); Abdollahi et al. (2015); Ivanauskas et al. (2014a). KauD14; VilnD06 (Ivanauskas et al. 2014b). Akm01; AlytD01; AlytD02; AlytD09; Kal02; KauD24; Kėd02; Kel03; Kel05; KlaiD02; Rok02; Rok07; Rok08; Šiau02; Šiau06; Šilu13; Šven01; Šven08; Šven16; Šven29; Šven30; Var28.

#### *Graphocephala* Van Duzee, 1916

* ***Graphocephala fennahi* Young, 1977**

iNaturalist record: VilnC10, 04 Aug. 2023 (iN176591036).

**Remarks**. A Nearctic species introduced to Europe in the 1930s (Nickel 2003). It occurs in parks, mainly on *Rhododendron* species (Nickel 2003; Gębicki et al. 2013; Zhuravleva et el. 2023). It is now widespread in Europe (Dmitriev et al. 2022). Records are known from countries neighbouring Lithuania. In particular, it was discovered in Poland in 1996 in Kórnik near Poznań (Gębicki et al. 2013) and in Latvia in 2016 in Riga (Piterāns 2016). In Lithuania, it is currently known only from an observation in Vilnius submitted to iNaturalist.

### Typhlocybinae Kirschbaum, 1868

**Alebrini McAtee, 1926**

#### *Alebra* Fieber, 1872

***Alebra albostriella* (Fallén, 1826)**: Vilbaste (1974); Nast (1987); Anufriev and Kirillova (1998); Demir (2008); Söderman et al. (2009). AlytD04; Ign09; Laz05; Rok09; Šila01; Šilu10; Vilk01.

***Alebra neglecta* Wagner, 1940**: Nast (1987); Söderman et al. (2009); Laz06; Var26 (Vilbaste 1974).

***Alebra wahlbergi* (Boheman, 1845)**: Vilbaste (1974); Ossiannilsson (1981); Nast (1987); Söderman et al. (2009). El02; Mar01; PanD01; PanD05.

### Dikraneurini McAtee, 1926

#### *Dikraneura* Hardy, 1850

***Dikraneura variata* Hardy, 1850**: Vilbaste (1974); Nast (1987); Anufriev and Kirillova (1998); Söderman et al. (2009). Ign06; PalM03; Šven24.

#### *Emelyanoviana* Anufriev, 1970

***Emelyanoviana mollicula* (Boheman, 1845)**: Vilbaste (1974); Nast (1987); Anufriev and Kirillova (1998); Söderman et al. (2009); Nickel et al. (2014). Šilu10.

#### *Erythria* Fieber, 1866

***Erythria aureola* (Fallén, 1806)**: Vilbaste (1974); Ossiannilsson (1981); Nast (1987); Söderman et al. (2009). Laz06; Ras01.

#### *Forcipata* DeLong & Caldwell, 1942

***Forcipata citrinella* (Zetterstedt, 1828)**: Vilbaste (1974); Nast (1987); Anufriev and Kirillova (1998); Söderman et al. (2009). Bir01; Kel03; Kel05; Šiau02; Šiau06; Šven17.

***Forcipata forcipata* (Flor, 1861)**: Vilbaste (1974); Ossiannilsson (1981); Nast (1987); Anufriev and Kirillova (1998); Söderman et al. (2009).

#### *Micantulina* Anufriev, 1970

***Micantulina pseudomicantula* (Knight, 1965)**: Ossiannilsson (1981); Nast (1987); Söderman et al. (2009); Guglielmino et al. (2024); Kwon et al. (2025). KauD01 (Vilbaste 1974).

#### *Notus* Fieber, 1866

***Notus flavipennis* (Zetterstedt, 1828)**: Vilbaste (1974); Nast (1987); Anufriev and Kirillova (1998); Söderman et al. (2009). VilnD06 (Ivanauskas et al. 2014b). AlytD01; AlytD02; Ign09; Kel05; KlaiD02; KlaiD07; Šilu04; Šilu05; Šilu10; Šilu11; Šilu12; Šven01; Šven06; Šven16; Šven18; Šven29; Trak03.

#### *Vilbasteana* Anufriev, 1970

***Vilbasteana oculata* (Lindberg, 1929)** (= *Igutettix oculatus* (Lindberg, 1929)): Kwon et al. (2025). Anyk03 (Stalažs (2013)). KauC07; Pag01.

#### *Wagneriala* Anufriev, 1970

***Wagneriala minima* (Sahlberg, 1871)**: Vilbaste (1974); Ossiannilsson (1981); Nast (1987); Söderman et al. (2009). Anyk01; Ign06; Var17.

### Empoascini Distant, 1908

#### *Austroasca* Lower, 1952

***Austroasca vittata* (Lethierry, 1884)**: Söderman et al. (2009); Lu and Qin (2014). PanD01; Ras01; Šven24 (Vilbaste 1974). Jurb02.

#### *Chlorita* Fieber, 1872

***Chlorita dumosa* (Ribaut, 1933)**: Ossiannilsson (1981); Nast (1987); Söderman et al. (2009). Kaiš07; Trak18 (Vilbaste 1974).

***Chlorita paolii* (Ossiannilsson, 1939)**: Vilbaste (1974); Ossiannilsson (1981); Nast (1987); Anufriev and Kirillova (1998); Demir (2008); Söderman et al. (2009). KauD14 (Ivanauskas et al. 2014b). AlytD08; Laz06; Rok05; Šak04; Šven03; Var36; Vilk01.

#### *Hebata* DeLong, 1931

***Hebata affinis* (Nast, 1937)**: Nast (1987); Söderman et al. (2009). Var01 (Söderman and Dapkus 2009). PanD01.

***Hebata decipiens* (Paoli, 1930)** (= *Empoasca decipiens* Paoli, 1930): Nast (1987); Demir (2008); Söderman et al. (2009). AlytC01; Ign04; PanD01; Šven24 (Vilbaste 1974). PanD05.

***Hebata solani* (Curtis, 1846)** (= *Empoasca solani* (Curtis, 1846)): Vilbaste (1974); Nast (1987); Söderman et al. (2009). PanC01 (Mensonienė 1979). AlytD02; Ign09; Ner01; PanD01; PanD05; Plu03; Šiau02; Šilu01; Šilu10; Šilu12; Vilk01; VilnD04.

***Hebata vitis* (Göthe, 1875)** (= *Empoasca vitis* (Göthe, 1875)): Vilbaste (1974); Nast (1987); Söderman et al. (2009). PanC01 (Mensonienė 1979); VilnD03; VilnD06 (Ivanauskas et al. 2014b). AlytD02; Kaiš07; Ner04; PanD04; PanD05; Šilu12.

#### *Kybos* Fieber, 1866

***Kybos abstrusus* (Linnavuori, 1950)**: Ossiannilsson (1981); Nast (1987); Mühlethaler et al. (2009); Söderman et al. (2009); Mitjaev (2002, 2015). Rad04 (Vilbaste 1974).

***Kybos butleri* (Edwards, 1908)**: Vilbaste (1974); Ossiannilsson (1981); Nast (1987); Anufriev and Kirillova (1998); Mühlethaler et al. (2009); Söderman et al. (2009). Mar01.

***Kybos oshanini* Zakhvatkin, 1953**: Vilbaste (1974); Nast (1987). Jurb02; Ras04.

***Kybos populi* (Edwards, 1908)**: Söderman et al. (2009). Trak07 (Söderman and Rintala 2009).

***Kybos smaragdula* (Fallén, 1806)**: Vilbaste (1974); Nast (1987); Anufriev and Kirillova (1998); Mühlethaler et al. (2009); Söderman et al. (2009). Jurb02; Ras01.

***Kybos virgator* (Ribaut, 1933)**: Vilbaste (1974); Nast (1987); Demir (2008); Mühlethaler et al. (2009); Söderman et al. (2009). PanD04; Šven24.

### Typhlocybini Kirschbaum, 1868

#### *Aguriahana* Distant, 1918

***Aguriahana germari* (Zetterstedt, 1837)** (= *Wagneripteryx germari* (Zetterstedt, 1840)): Vilbaste (1974); Nast (1987); Anufriev and Kirillova (1998); Söderman et al. (2009). AlytD05; El02; PanD01; Šak03; Šven03; Vilk01; Vilk01.

***Aguriahana pictilis* (Stål, 1853)**: Vilbaste (1974); Ossiannilsson (1981); Nast (1987); Söderman et al. (2009).

***Aguriahana stellulata* (Burmeister, 1841)**: Ossiannilsson (1981); Nast (1987); Anufriev and Kirillova (1998); Söderman et al. (2009); Laz05 (Vilbaste 1974).

#### *Edwardsiana* Zakhvatkin, 1929

***Edwardsiana ampliata* (Wagner, 1947)**: Söderman et al. (2009). Trak06 (Söderman and Rintala 2009).

***Edwardsiana avellanae* (Edwards, 1888)** (= *E. staminata* (Ribaut, 1931)): Ossiannilsson (1981); Nast (1987); Söderman et al. (2009). PanD01; PanD05 (Vilbaste 1974).

***Edwardsiana bergmani* (Tullgren, 1916)**: Vilbaste (1974); Niedringhaus and Olthoff (1993); Söderman et al. (2009). Var01 (Söderman and Dapkus 2009). AlytD02; Jona01.

***Edwardsiana candidula* (Kirschbaum, 1868)**: Ossiannilsson (1981); Nast (1987); Söderman et al. (2009). PanD01 (Vilbaste 1974).

***Edwardsiana crataegi* (Douglas, 1876)**: Var01 (Söderman and Dapkus 2009).

***Edwardsiana flavescens* (Fabricius, 1794)**: Ossiannilsson (1981); Nast (1987); Söderman et al. (2009). AlytC01 (Vilbaste 1974).

***Edwardsiana frustrator* (Edwards, 1908)**: Söderman et al. (2009). Trak01; Trak08; Var17 (Söderman and Rintala 2009).

***Edwardsiana geometrica* (Schrank, 1801)**: Vilbaste (1974); Ossiannilsson (1981); Nast (1987); Anufriev and Kirillova (1998); Söderman et al. (2009). AlytD04; Ign09; Laz05; Mar01; Mol04; PanD01; Rok04; Trak03; Var12.

***Edwardsiana gratiosa* (Boheman, 1852)**: Ossiannilsson (1981); Nast (1987); Söderman et al. (2009). Laz05; VilnD07 (Vilbaste 1974). VilnD11.

***Edwardsiana lethierryi* (Edwards, 1881)**: Söderman et al. (2009). Vilk01 (Vilbaste 1974); Trak01 (Söderman and Rintala 2009).

***Edwardsiana menzbieri* Zakhvatkin, 1948**: Vilbaste (1974); Ossiannilsson (1981); Nast (1987); Anufriev and Kirillova (1998); Söderman et al. (2009). Plu04; Ras04; Šilu02; Šven03; Telš02; VilnD04.

***Edwardsiana plebeja* (Edwards, 1914)**: Ossiannilsson (1981); Nast (1987); Söderman et al. (2009). PanD05 (Vilbaste 1974).

***Edwardsiana plurispinosa* (Wagner, 1935)**: Söderman et al. (2009). Trak06 (Söderman and Rintala 2009).

***Edwardsiana prunicola* (Edwards, 1914)**: Ossiannilsson (1981); Nast (1987); Söderman et al. (2009). VilnD05 (Vilbaste 1974). AlytD02; KlaiD02; Ras04.

***Edwardsiana rosae* (Linnaeus, 1758)**: Söderman et al. (2009). PanC01 (Mensonienė 1979).

***Edwardsiana salicicola* (Edwards, 1885)**: Ossiannilsson (1981); Nast (1987); Söderman et al. (2009). PanD01; Pas01; Vilk01 (Vilbaste 1974). AlytD02; Ign09; Šven25.

***Edwardsiana sociabilis* (Ossiannilsson, 1936)**: PanC01 (Mensonienė 1979).

***Edwardsiana soror* (Linnavuori, 1950)**: Vilbaste (1974); Ossiannilsson (1981); Nast (1987); Söderman et al. (2009). Šilu02; VilnD04; VilnD07.

***Edwardsiana tersa* (Edwards, 1914)**: Ossiannilsson (1981); Nast (1987); Anufriev and Kirillova (1998); Söderman et al. (2009); Thanou et al. (2018). Jurb02 (Vilbaste 1974).

***Edwardsiana ulmiphagus* Wilson & Claridge, 1999**: Söderman et al. (2009).

#### *Eupterycyba* Dlabola, 1958

***Eupterycyba jucunda* (Herrich-Schäffer, 1837)**: Ossiannilsson (1981); Nast (1987); Söderman et al. (2009). Laz05; VilnD11 (Vilbaste 1974). Šilu12.

#### *Eupteryx* Curtis, 1831

***Eupteryx adspersa* (Herrich-Schäffer, 1838)**: Söderman et al. (2009). PanD01 (Vilbaste 1974).

***Eupteryx atropunctata* (Goeze, 1778)**: Vilbaste (1974); Nast (1987); Anufriev and Kirillova (1998); Söderman et al. (2009); Nickel et al. (2014). VilnD03 (Ivanauskas et al. 2014b). AlytD01; AlytD02; KlaiD07; PanD04; Plu03; Ras04; Šilu05; Šilu10; Šilu11; Šilu12; Tau03; Vilk01.

***Eupteryx aurata* (Linnaeus, 1758)**: Vilbaste (1974); Anufriev and Kirillova (1998); Söderman et al. (2009); Nickel et al. (2014). AlytD02; Mar01; Mol01; Ner02; Ner04; PanD05; Plu04; Šilu01; Tau03; Var12; Vilk01.

***Eupteryx calcarata* Ossiannilsson, 1936**: Vilbaste (1974); Ossiannilsson (1981); Nast (1987); Anufriev and Kirillova (1998); Söderman et al. (2009). Šven24.

***Eupteryx collina* (Flor, 1861)**: Nast (1987); Söderman et al. (2009). Laz06; Šven03 (Vilbaste 1974). Kaiš07; Plu04; Ras02; Ras05; Šven07.

***Eupteryx cyclops* Matsumura, 1906**: Vilbaste (1974); Ossiannilsson (1981); Nast (1987); Anufriev and Kirillova (1998); Söderman et al. (2009). AlytD02; Ign09; Plu04; Šilu01; Šilu10; Šilu12; Tau03.

***Eupteryx notata* Curtis, 1837**: Vilbaste (1974); Nast (1987); Anufriev and Kirillova (1998); Söderman et al. (2009). PanC01 (Mensonienė 1979). AlytD02; KlaiD07; PanD05; Šilu04.

***Eupteryx origani* Zakhvatkin, 1948**: Vilbaste (1974); Ossiannilsson (1981); Nast (1987); Guglielmino et al. (2005); Söderman et al. (2009). B01; Šilu01; Šven24; Tau02; Vilk01.

***Eupteryx signatipennis* (Boheman, 1847)**: Söderman et al. (2009). Trak06 (Söderman and Rintala 2009).

***Eupteryx stachydearum* (Hardy, 1850)**: Vilbaste (1974); Nast (1987); Anufriev and Kirillova (1998); Söderman et al. (2009).

***Eupteryx tenella* (Fallén, 1806)**: Vilbaste (1974); Ossiannilsson (1981); Nast (1987); Anufriev and Kirillova (1998); Söderman et al. (2009). Jurb02; Kaiš07; PanD02; Vilk01.

***Eupteryx urticae* (Fabricius, 1803)**: Vilbaste (1974); Le Quesne (1976); Nast (1987); Söderman et al. (2009). Ner02; Ner04; Plu04; Ras01; Šilu12.

***Eupteryx vittata* (Linnaeus, 1758)**: Vilbaste (1974); Nast (1987); Anufriev and Kirillova (1998); Söderman et al. (2009). AlytD02; Mar01; Mol03; Ner02; Ner04; Plu04; Ras01; Rok03; Šilu10; Šilu11; Šilu12; Vilk01.

#### *Eurhadina* Haupt, 1929

***Eurhadina concinna* (Germar, 1831)**: Ossiannilsson (1981); Nast (1987); Anufriev and Kirillova (1998); Söderman et al. (2009). AlytC01; AlytD04; AlytD05; Šven03 (Vilbaste 1974).

***Eurhadina kirschbaumi* Wagner, 1937**: Vilbaste (1974); Ossiannilsson (1981); Nast (1987); Anufriev and Kirillova (1998); Söderman et al. (2009).

***Eurhadina pulchella* (Fallén, 1806)**: Vilbaste (1974); Nast (1987); Anufriev and Kirillova (1998); Söderman et al. (2009). PanC01 (Mensonienė 1979). AlytD02; AlytD05; El02; Ign09; Kal03; Laz05; PanD05; Telš02; Vilk01.

***Eurhadina saageri* Wagner, 1937**: Vilbaste (1974); Nast (1987); Anufriev and Kirillova (1998); Söderman et al. (2009). AlytD05; Kal03; Laz05; PanD05; Vilk01.

#### *Fagocyba* Dlabola, 1958

***Fagocyba carri* (Edwards, 1914)**: Vilbaste (1974); Ossiannilsson (1981); Nast (1987); Söderman et al. (2009). El02; PanD05; Vilk01.

***Fagocyba cruenta* (Herrich-Schäffer, 1838)**: Nast (1987); Söderman et al. (2009). AlytC01; AlytD04; El02; PanD05; Vilk01 (Vilbaste 1974).

#### *Linnavuoriana* Dlabola, 1958

***Linnavuoriana decempunctata* (Fallén, 1806)**: Söderman et al. (2009). Var01 (Söderman and Dapkus 2009).

***Linnavuoriana sexmaculata* (Hardy, 1850)**: Vilbaste (1974); Nast (1987); Anufriev and Kirillova (1998); Söderman et al. (2009). AlytD02; Ign09; Jurb03; PanD01; PanD02; Pas01; Rok04; Šiau06; Šilu12; Trak18.

#### *Ribautiana* Zakhvatkin, 1947

***Ribautiana tenerrima* (Herrich-Schäffer, 1834)**: Söderman et al. (2009). Trak01 (Söderman and Rintala 2009).

***Ribautiana ulmi* (Linnaeus, 1758)**: Vilbaste (1974); Nast (1987); Anufriev and Kirillova (1998); Söderman et al. (2009). AlytD02.

#### *Typhlocyba* Germar, 1833

***Typhlocyba quercus* (Fabricius, 1777)**: Vilbaste (1974); Nast (1987); Anufriev and Kirillova (1998); Söderman et al. (2009). Ign09; KauD01; Laz05; PanD05; Var12; Vilk01; VilnD11.

#### *Zonocyba* Vilbaste, 1982

***Zonocyba bifasciata* (Boheman, 1851)** (= *Typhlocyba bifasciata* Boheman, 1851): Nast (1987); Söderman et al. (2009). AlytD04 (Vilbaste 1974).

### Erythroneurini Young, 1952

#### *Alnetoidia* Dlabola, 1958

***Alnetoidia alneti* (Dahlbom, 1850)**: Vilbaste (1974); Nast (1987); Anufriev and Kirillova (1998); Söderman et al. (2009). PanD04; Šilu10; Šilu12.

#### *Arboridia* Zakhvatkin, 1946

***Arboridia parvula* (Boheman, 1845)**: Vilbaste (1974); Nast (1987); Anufriev and Kirillova (1998). Kel03; Kel05; Laz06; Rok09.

***Arboridia ribauti* (Ossiannilsson, 1937)**: Nast (1987); Söderman et al. (2009). El02; PanD05 (Vilbaste 1974).

***Arboridia velata* (Ribaut, 1952)**: Nast (1987); Anufriev and Kirillova (1998); Söderman et al. (2009). El02 (Vilbaste 1974).

#### *Zygina* Fieber, 1866

***Zygina angusta* Lethierry, 1874**: Söderman et al. (2009). Trak05 (Söderman and Rintala 2009).

***Zygina flammigera* (Fourcroy, 1785)** (= *Flammigeroidia flammigera* (Fourcroy, 1785)): Vilbaste (1974); Nast (1987); Anufriev and Kirillova (1998); Söderman et al. (2009). VilnD03 (Ivanauskas et al. 2014b). PanD01; PanD05; Vilk01.

***Zygina hyperici* (Herrich-Schäffer, 1836)** (= *Hypericiella hyperici* (Herrich-Schäffer, 1836)): Vilbaste (1974); Nast (1987); Anufriev and Kirillova (1998); Söderman et al. (2009). Plu03; Šven03; Trak15; Ute03; VilnD07.

***Zygina ordinaria* (Ribaut, 1936)** (= *Flammigeroidia ordinaria* (Ribaut, 1936)): Nast (1987); Demir (2008); Söderman et al. (2009). Trak03 (Vilbaste 1974). AlytC01; Pas01; Šven03; Vilk01; VilnD11.

***Zygina rubrovittata* (Lethierry, 1869)** (= *Flammigeroidia rubrovittata* (Lethierry, 1869)): Nast (1987); Söderman et al. (2009). VilnD07 (Vilbaste 1974).

***Zygina suavis* Rey, 1891** (= *Flammigeroidia suavis* (Rey, 1891)): Vilbaste (1974); Nast (1987); Söderman et al. (2009).

***Zygina tiliae* (Fallén, 1806)** (= *Flammigeroidia tiliae* (Fallén, 1806)): Vilbaste (1974); Nast (1987); Söderman et al. (2009).

#### *Zyginidia* Haupt, 1929

*Zyginidia scutellaris (Herrich-Schäffer, 1838) – Šilu12: 1♂ (MZVUHe01908), 3–15 Jul. 2024, Malaise trap, A. Petrašiūnas leg.; KauD02: Braziūkai, 54.90144, 23.48473, 1♂, 5♀, 06 Nov. 2025, grassland on sandy soil, V. Tamutis leg.

**Remarks**. The distribution of the species requires clarification. In particular, the species has not been recorded at its type locality for approximately 20 years. It has also been noted that *Z. scutellaris* has been recorded in Germany since the 1950s; however, in specific habitats, the species’ absence was reliably confirmed during the 1960s and 1970s (Nickel 2003). Among the countries neighbouring Lithuania, it is known only from Poland, where it is a rare species associated with the Baltic coast (Gębicki et al. 2013). We have also recorded the species in Poland (Warmian-Masurian Voivodeship, Mrągowo County, Gmina Mikołajki, Urwitałt environs) (TUZ020040, TUZ020181). There are also records from Estonia (Remm 1970), which require confirmation.

***Zyginidia viaduensis* (Wagner, 1941)**: Nast (1987); Söderman et al. (2009). Anyk01 (Vilbaste 1974).

### Deltocephalinae Fieber, 1869

**Eupelicini Sahlberg, 1871**

#### *Eupelix* Germar, 1821

***Eupelix cuspidata* (Fabricius, 1775)**: Vilbaste (1974); Nast (1987); Söderman et al. (2009); Abdollahi et al. (2015). Kėd01; Rad04; Trak15; Ute03; Var36; Vilk01; VilnC07.

### Opsiini Emeljanov, 1962

#### *Neoaliturus* Distant, 1918

***Neoaliturus fenestratus* (Herrich-Schäffer, 1834)**: Vilbaste (1974); Nast (1987); Demir (2008); Söderman et al. (2009); Abdollahi et al. (2015). Ign06; Šven24; Trak18; Ute01; Var36; Var37; VilnD07.

***Neoaliturus guttulatus* (Kirschbaum, 1868)**: Vilbaste (1974). B01; Drus01; Šven03; Var36; Var37; VilnD07.

### Macrostelini Kirkaldy, 1906

#### *Balclutha* Kirkaldy, 1900

***Balclutha arhenana* Dlabola, 1967**: Söderman et al. (2009). Var01 (Söderman and Dapkus 2009).

***Balclutha boica* Wagner, 1950**: Nickel (2003); Söderman et al. (2009). K.R.01.

***Balclutha calamagrostis* Ossiannilsson, 1961**: Vilbaste (1974); Ossiannilsson (1983); Nast (1987); Söderman et al. (2009). VilnD06 (Ivanauskas et al. 2014b). AlytD09; Var37; VilnD04.

***Balclutha lineolata* (Horváth, 1904)**: Vilbaste (1974); Nast (1987).

***Balclutha punctata* (Fabricius, 1775)**: Vilbaste (1974); Söderman et al. (2009); Abdollahi et al. (2015). PanC01 (Mensonienė 1979); KauD14 (Ivanauskas et al. 2014b). Drus01; Kel03; PanD04; Plu03; Rok09; Šilu02; Vilk01; Zar02.

#### *Coryphaelus* Puton, 1886

***Coryphaelus gyllenhalii* (Fallén, 1826)** (= *Coryphaeus gyllenhalii* (Fallén, 1826)): Nast (1987); Söderman et al. (2009). Laz06 (Vilbaste 1974).

#### *Macrosteles* Fieber, 1866

***Macrosteles alpinus* (Zetterstedt, 1828)**: Kwon and Know (2022). Šiau04.

***Macrosteles cristatus* (Ribaut, 1927)**: Vilbaste (1974); Nast (1987); Söderman et al. (2009); Kwon and Kwon (2022). PanC01 (Mensonienė 1979); VilnD06 (Ivanauskas et al. 2014b). Tau02.

***Macrosteles frontalis* (Scott, 1875)**: Vilbaste (1974); Nast (1987); Söderman et al. (2009); Kwon and Kwon (2022); Ras04; Šiau06.

***Macrosteles horvathi* (Wagner, 1935)**: Vilbaste (1974); Söderman et al. (2009); Kwon and Kwon (2022). Plu03.

***Macrosteles laevis* (Ribaut, 1927)**: Vilbaste (1974); Nast (1987); Demir (2008); Söderman et al. (2009); Abdollahi et al. (2015); Kwon and Kwon (2022). AlytD02; Anyk06; Ign07; Kaiš07; Kel03; Kel05; Ner01; PanD02; Plu03; Ras01; Rok01; Rok04; Rok05; Šiau02; Šiau06; Šven12; Šven24; Šven28; Var36; Var37; Vilk01; VilnD04.

***Macrosteles lividus* (Edwards, 1894)**: Nast (1987); Söderman et al. (2009); Kwon and Kwon (2022). Laz06; Var17; Var36 (Vilbaste 1974).

***Macrosteles nubilus* (Ossiannilsson, 1936)**: Söderman et al. (2009); Kwon and Kwon (2022). Var01 (Söderman and Dapkus 2009).

***Macrosteles oshanini*Razvyazkina, 1957**: Nast (1987); Söderman et al. (2009); Kwon and Kwon (2022). Anyk02 (Vilbaste 1974).

***Macrosteles ossiannilssoni* Lindberg, 1954**: Vilbaste (1974); Nast (1987); Söderman et al. (2009); Kwon and Kwon (2022). B01; PanD02.

***Macrosteles pygmaeus* Vilbaste, 1974**: Nast 1982, 1987; Kwon and Kwon (2022). Šven14; Zar01 (Vilbaste 1974). Šven01.

***Macrosteles quadripunctulatus* (Kirschbaum, 1868)**: Vilbaste (1974); Nast (1987); Demir (2008); Söderman et al. (2009); Kwon and Kwon (2022). Ign04; Var37; Vilk01.

***Macrosteles septemnotatus* (Fallén, 1806)**: Vilbaste (1974); Nast (1987); Söderman et al. (2009); Kwon and Kwon (2022). K.R.01; Mol03; Šven03.

***Macrosteles sexnotatus* (Fallén, 1806)**: Vilbaste (1974); Nast (1987); Söderman et al. (2009); Ivanauskas et al. (2011, 2014a); Abdollahi et al. (2015); Kwon and Kwon (2022). KauD14; VilnD03 (Ivanauskas et al. 2014b). Plu04; Ras04; Šilu02; Tau02; Tau03; Telš02.

***Macrosteles variatus* (Fallén, 1806)**: Vilbaste (1974); Nast (1987); Söderman et al. (2009); Kwon and Kwon (2022). AlytD02; Mol03; PalM03; PanC01.

***Macrosteles viridigriseus* (Edwards, 1924)**: Vilbaste (1974); Nast (1987); Anufriev and Kirillova (1998); Söderman et al. (2009); Kwon and Kwon (2022). Anyk02.

#### *Sagatus* Ribaut, 1948

***Sagatus punctifrons* (Fallén, 1826)**: Vilbaste (1974); Nast (1987); Anufriev and Kirillova (1998); Söderman et al. (2009); Kwon and Kwon (2022). KauD14 (Ivanauskas et al. 2014b). Jurb04; Kel05; Rok04; Ute05.

#### *Sonronius* Dorst, 1937

***Sonronius binotatus* (Sahlberg, 1871)**: Nast (1987); Söderman et al. (2009); Kwon and Kwon (2022). VilnD07 (Vilbaste 1974). Šven28.

***Sonronius dahlbomi* (Zetterstedt, 1837)**: Vilbaste (1974); Nast (1987); Söderman et al. (2009).

### Deltocephalini Fieber, 1869

#### *Deltocephalus* Burmeister, 1838

***Deltocephalus pulicaris* (Fallén, 1806)**: Vilbaste (1974); Nast (1987); Söderman et al. (2009). PanC01 (Mensonienė 1979). Šilu10; Šilu11; Šven17; Var36.

### Chiasmini Distant, 1908

#### *Doratura* Sahlberg, 1871

***Doratura exilis* Horváth, 1903**: Vilbaste (1974); Nast (1987); Söderman et al. (2009); Bückle and Guglielmino (2022). Anyk01; Ign06; Šven24; Var17; Var37.

***Doratura homophyla* (Flor, 1861)** (= *Doratura littoralis* Kuntze, 1937): Metcalf (1967); Vilbaste (1974); Nast (1987); Demir (2008); Söderman et al. (2009); Bückle and Guglielmino (2022). Trak12; Trak15; Ute03; Var36.

***Doratura impudica* Horváth, 1897**: Vilbaste (1974); Nast (1987); Söderman et al. (2009); Bückle and Guglielmino (2022). Trak15; Ute03.

***Doratura stylata* (Boheman, 1847)**: Metcalf (1967); Vilbaste (1974); Nast (1987); Söderman et al. (2009); Abdollahi et al. (2015); Bückle and Guglielmino (2022). Jurb02; Mar01; Mol03; Rok09; Šilu12; Šven08; Šven29; Trak12; Var36; Zar02.

### Fieberiellini Wagner, 1951

#### Fieberiella Signoret, 1880

***Fieberiella macchiae* Linnavuori, 1962**: PanC01 (Mensonienė 1979).

***Fieberiella septentrionalis* Wagner, 1963**: Söderman et al. (2009). Šven04; Trak06; Trak07; Trak19 (Söderman and Rintala 2009). Šak06; Šak07; Šak10; Šak11; Širv01.

### Athysanini Van Duzee, 1892

#### *Allygidius* Ribaut, 1948

***Allygidius commutatus* (Fieber, 1872)**: Vilbaste (1974); Nast (1987); Söderman et al. (2009). AlytC01; Ign06; Laz06; Ner01; Ner04; PanD01; Šven03; Šven14; Zar03.

#### *Allygus* Fieber, 1872

***Allygus communis* (Ferrari, 1882)**: Söderman et al. (2009) Jurb06; Kal02; Ner07; Šak12.

***Allygus mixtus* (Fabricius, 1794)**: Vilbaste (1974); Nast (1987); Söderman et al. (2009); PanC01 (Mensonienė 1979). AlytC01; AlytD05; Bir01; Jurb06; Kaiš06; KauC01; PanD01; Šak08; Šak12; Trak03; Zar03.

***Allygus modestus* Scott, 1876**: Söderman et al. (2009).

#### *Athysanus* Burmeister, 1838

***Athysanus argentarius* Metcalf, 1955**: Vilbaste (1974); Nast (1987); Söderman et al. (2009). PanC01 (Mensonienė 1979). AlytD01; K.R.03; KauD24; KlaiD02; KlaiD07; Pag01; Ute01; Var28; Vilk01.

***Athysanus quadrum* Boheman, 1845**: Vilbaste (1974); Nast (1987); Anufriev and Kirillova (1998); Guglielmino et al. (2005); Söderman et al. (2009); Trivellone et al. (2015). AlytD02; AlytD05; AlytD09; Ign10; Pag01; Telš03; Ute01.

#### *Conosanus* Osborn & Ball, 1902

***Conosanus obsoletus* (Kirschbaum, 1858)**: Vilbaste (1974); Nast (1987); Söderman et al. (2009). Mar01; PanD05; Šila01.

#### *Doliotettix* Ribaut, 1942

***Doliotettix lunulatus* (Zetterstedt, 1837)**: Vilbaste (1974); Nast (1987); Söderman et al. (2009).

#### *Euscelidius* Ribaut, 1942

***Euscelidius schenckii* (Kirschbaum, 1868)**: Nast (1987); Anufriev and Kirillova (1998); Söderman et al. (2009). VilnC09 (Vilbaste 1974). Širv01; Šven16.

#### *Euscelis* Brullé, 1832

***Euscelis distinguenda* (Kirschbaum, 1858)**: Vilbaste (1974); Nast (1987); Anufriev and Kirillova (1998); Söderman et al. (2009); Khoobdel and Palarpour-Rayeni (2021). Ign06; Laz06; Šven03; Šven14; Šven24; Var36; Var37.

***Euscelis incisa* (Kirschbaum, 1858)**: Vilbaste (1974); Nast (1987); Söderman et al. (2009); Ivanauskas et al. (2011, 2014a); Khoobdel Khoobdel and Palarpour-Rayeni (2021). KauD14 (Ivanauskas et al. 2014b). Šilu10; Var36.

#### *Graphocraerus* Thomson, 1869

***Graphocraerus ventralis* (Fallén, 1806)**: Vilbaste (1974); Nast (1987); Söderman et al. (2009). PanC01 (Mensonienė 1979); KauD14 (Ivanauskas et al. 2014b). Bir01; KlaiD07; Šilu05; Šilu10; Šven19; Šven28; Šven29; Trak12.

#### *Grypotes* Fieber, 1866

***Grypotes puncticollis* (Herrich-Schäffer, 1834)**: Vilbaste (1974); Nast (1987); Söderman et al. (2009). El02; PanD01; Šak03; Šven01; Trak18; Vilk01.

#### *Handianus* Ribaut, 1942

***Handianus flavovarius* (Herrich-Schäffer, 1835)**: Nast (1987); Nickel (2003); Söderman et al. (2009). Ras01 (Vilbaste 1974). KauD04; Šven28; Šven29; Trak12.

#### *Hesium* Ribaut, 1942

***Hesium domino* (Reuter, 1880)**: Vilbaste (1974); Nast (1987); Söderman et al. (2009). AlytD04; Anyk02; Jurb07; Kaiš06; KauD19; PanD01; Plu03; Šak03; Šila01; Šven24; Šven28; Šven29; Trak18; VilnD07.

#### *Idiodonus* Ball, 1936

***Idiodonus cruentatus* (Panzer, 1799)**: Vilbaste (1974); Nast (1987); Söderman et al. (2009). Šven24.

#### *Laburrus* Ribaut, 1942

***Laburrus impictifrons* (Boheman, 1852)**: Vilbaste (1974); Söderman et al. (2009). Ign06; Rok04; Šven03; Šven28; Šven29.

#### *Lamprotettix* Ribaut, 1952

***Lamprotettix nitidulus* (Fabricius, 1787)**: Nast (1987); Söderman et al. (2009). Laz02; PanD04; Var26 (Vilbaste 1974). Kaiš06; Kre02.

#### *Macustus* Ribaut, 1942

***Macustus grisescens* (Zetterstedt, 1828)**: Vilbaste (1974); Nast (1987); Söderman et al. (2009). KlaiD02; Šven17; Šven28; Trak12; Trak13.

#### *Pithyotettix* Ribaut, 1942

***Pithyotettix abietinus* (Fallén, 1806)**: Vilbaste (1974); Nast (1987); Anufriev and Kirillova (1998); Söderman et al. (2009). B01; Ner06.

#### *Speudotettix* Ribaut, 1942

***Speudotettix subfusculus* (Fallén, 1806)**: Vilbaste (1974); Nast (1987); Söderman et al. (2009). Drus01; Plu03; Plu04; Rad05; Ras05; Rok03.

#### *Stictocoris* Thomson, 1869

***Stictocoris picturatus* (Sahlberg, 1842)**: Nast (1987); Söderman et al. (2009). Šven24 (Vilbaste 1974). Šven28; Trak12; Trak14.

#### *Streptanus* Ribaut, 1942

***Streptanus aemulans* (Kirschbaum, 1868)**: Vilbaste (1974); Nast (1987); Söderman et al. (2009). PanC01 (Mensonienė 1979). Bir01; Pas01; Šilu11; Trak12; Vilk01.

***Streptanus confinis* (Reuter, 1880)**: Nast (1987); Anufriev and Kirillova (1998); Söderman et al. (2009). Bir01; K.R.01 (Vilbaste 1974). Kup02; Laz05; Pag01.

***Streptanus marginatus* (Kirschbaum, 1858)**: Vilbaste (1974); Nast (1987); Söderman et al. (2009). Šven14.

***Streptanus sordidus* (Zetterstedt, 1828)**: Vilbaste (1974); Nast (1987); Söderman et al. (2009); Ign04; Kal02; Laz05; Telš03.

### Thamnotettix Zetterstedt, 1837

***Thamnotettix confinis* (Zetterstedt, 1828)**: Vilbaste (1974); Nast (1987); Söderman et al. (2009). Ign04; PanC01; PanD04; Šven14; Zar03.

### Platymetopiini Haupt, 1929

#### *Colladonus* Ball, 1936

***Colladonus torneella* (Zetterstedt, 1828)**: Vilbaste (1974); Nast (1987); Anufriev and Kirillova (1998); Söderman et al. (2009). AlytD08.

#### *Platymetopius* Burmeister, 1838

***Platymetopius guttatus* Fieber, 1869**: Söderman et al. (2009). Trak19 (Söderman and Rintala 2009).

***Platymetopius undatus* (De Geer, 1773)**: Vilbaste (1974); Nast (1987); Söderman et al. (2009). El02; Ign09; Kel03; Šiau06; Šven03; Trak18.

### Cicadulini Van Duzee, 1892

#### *Cicadula* Zetterstedt, 1837

***Cicadula albingensis* Wagner, 1940**: Nast (1987); Anufriev and Kirillova (1998); Söderman et al. (2009). PanD05 (Vilbaste 1974).

***Cicadula flori* (Sahlberg, 1871)**: Vilbaste (1974); Nast (1987); Anufriev and Kirillova (1998); Söderman et al. (2009). AlytD01; AlytD02; Ign09; Kel05; KlaiD02; KlaiD07; Šilu05; Šilu10; Šven25; VilnD04.

***Cicadula frontalis* (Herrich-Schäffer, 1835)**: Vilbaste (1974); Söderman et al. (2009).

***Cicadula longiventris* (Sahlberg, 1871)** (= *Cicadula rubroflava* Linnavuori, 1952): Vilbaste (1974); Nast (1987); Söderman et al. (2009). PanD01.

***Cicadula nigricornis* (Sahlberg, 1871)**: Vilbaste (1974); Nast (1987); Söderman et al. (2009). Šven03.

***Cicadula persimilis* (Edwards, 1920)**: Vilbaste (1974); Nast (1987); Anufriev and Kirillova (1998); Söderman et al. (2009). Mar01; Rok06; Vilk01.

***Cicadula quadripunctata* (De Villers, 1789)** (= *Cicadula quadrinotata* (Fabricius, 1794)): Vilbaste (1974); Nast (1987); Söderman et al. (2009). PanC01 (Mensonienė 1979). Mar01; PanD02; Rok06; Šilu05; Vilk01; VilnD04.

***Cicadula saturata* (Edwards, 1915)**: Vilbaste (1974); Nast (1987); Söderman et al. (2009). Kel05.

#### *Elymana* DeLong, 1936

***Elymana kozhevnikovi* (Zakhvatkin, 1938)** (= *Elymana ikumae* (Matsumura, 1911)): Vilbaste (1974); Nast (1987); Söderman et al. (2009); Naveed et al. (2023). Ign07; Šven03.

***Elymana sulphurella* (Zetterstedt, 1828)**: Vilbaste (1974); Nast (1987); Söderman et al. (2009); Naveed et al. (2023). PanC01 (Mensonienė 1979); KauD14; VilnD06 (Ivanauskas et al. 2014b). Bir04; Rok06; Šven29.

#### *Hardya* Edwards, 1922

***Hardya signifer* (Then, 1897)**: Nast (1987); Nickel (2003); Söderman et al. (2009). Var17; Var37 (Vilbaste 1974).

***Hardya tenuis* (Germar, 1821)**: Söderman et al. (2009). Var17 (Vilbaste 1974).

#### *Paluda* DeLong, 1937

*Paluda flaveola* (Boheman, 1845): Vilbaste (1974); Nast (1987); Anufriev and Kirillova (1998); Söderman et al. (2009). VilnC07.

#### *Rhopalopyx* Ribaut, 1939

***Rhopalopyx adumbrata* (Sahlberg, 1842)** (= *Paluda adumbrata* (Sahlberg, 1842)): Vilbaste (1974); Nast (1987); Söderman et al. (2009); Den Bieman et al. (2021). AlytD09; Laz05.

***Rhopalopyx preyssleri* (Herrich-Schäffer, 1838)** (= *Paluda preyssleri* (Herrich-Schäffer, 1838)): Vilbaste (1974); Nast (1987); Nast (1987); Söderman et al. (2009); Den Bieman et al. (2021). Mar01; Šven08; Trak12; Trak18; Vilk01.

***Rhopalopyx vitripennis* (Flor, 1861)** (= *Paluda vitripennis* (Flor, 1861)): Vilbaste (1974); Nast (1987); Söderman et al. (2009). Šven29; Trak18.

### Limotettigini Baker, 1915

#### *Limotettix* Sahlberg, 1871

***Limotettix atricapillus* (Boheman, 1845)**: Vilbaste (1973, 1974); Nast (1987); Söderman et al. (2009). AlytD02; AlytD09; Ute01; Var08.

***Limotettix ochrifrons* Vilbaste, 1973**: Vilbaste (1973, 1974); Anufriev (1978); Nast (1982, 1987); Söderman et al. (2009). Var08.

***Limotettix sphagneticus* Emeljanov, 1964**: Vilbaste (1973, 1974); Nast (1987); Söderman et al. (2009). Ign10; Trak03; Var08.

***Limotettix striola* (Fallén, 1806)**: Vilbaste (1974); Söderman et al. (2009); Abdollahi et al. (2015). Anyk02; Kėd01; KlaiD07; Telš05.

#### *Ophiola* Edwards, 1922

***Ophiola corniculus* (Marshall, 1866)** (= *Scleroracus corniculus* (Marshall, 1866)): Vilbaste (1974); Söderman et al. (2009); Nast (1987); Trivellone (2010).

***Ophiola decumana* (Kontkanen, 1949)** (= *Scleroracus decumanus* (Kontkanen, 1949)): Vilbaste (1974); Nast (1987); Söderman et al. (2009). Mol03; Šilu10; Šven08; Šven16; Šven24; Var36; Var37.

***Ophiola identica* (Tishechkin, 2003)**: Söderman et al. (2009).

***Ophiola russeola* (Fallén, 1826)** (= *Scleroracus plutonius* (Uhler, 1877), *S. russeolus* (Fallén, 1826)): Vilbaste (1974); Nast (1987); Söderman et al. (2009). Kel05; Laz06; Šilu07; Šven01; Šven06; Šven12; Šven14; Šven24; Šven26; Trak18; Var36.

***Ophiola transversa* (Fallén, 1826)** (= *Scleroracus transversus* (Fallén, 1826)): Vilbaste (1974); Nast (1987); Söderman et al. (2009). Šven24; Trak18; Var36.

### Paralimnini Distant, 1908

#### *Arocephalus* Ribaut, 1946

***Arocephalus languidus* (Flor, 1861)**: Nast (1987); Anufriev and Kirillova (1998); Söderman et al. (2009). Ras01 (Vilbaste 1974). AlytD02; Kup04; Pag01; Šilu05; Šven29.

***Arocephalus punctum* (Flor, 1861)**: Vilbaste (1974); Söderman et al. (2009). Šven14.

#### *Arthaldeus* Ribaut, 1946

*Arthaldeus arenarius Remane, 1960 – Pag01: 1♂ (MZVUHe00706), 12 Aug. 2022, yellow pan traps, J. Skuja leg.; Šven16: 1♂ (TUZ359695), 28 Jun. 2023, O. Borodin leg.; Šven28: 1♂ (TUZ359823), 28 Jun. 2023, meadow, O. Borodin leg.

**Remarks**. A monophagous species on *Calamagrostis epigejos* (Nickel 2003). For a long time, it was primarily recorded in the southern part of the Western Palearctic. During the last 20 years, it has been found in Belarus (Borodin 2004), England (Wilson et al. 2015), Finland (Albrecht et al. 2015), Latvia (Ziemelis 2012), and Poland (Gębicki et al. 2013).

***Arthaldeus pascuellus* (Fallén, 1826)**: Vilbaste (1974); Nast (1987); Söderman et al. (2009). KauD14 (Ivanauskas et al. 2014b). AlytD01; AlytD02; AlytD05; Ign02; Ign07; Ign09; Kal02; KauD10; Kel05; KlaiD02; Pag01; Šal01; Šilu04; Šilu05; Šilu10; Šilu11; Šilu12; Šven01; Šven08; Šven13; Šven16; Šven28; Šven29.

***Arthaldeus striifrons* (Kirschbaum, 1868)**: Vilbaste (1974); Nast (1987); Söderman et al. (2009). KauD14 (Ivanauskas et al. 2014b). AlytD02; AlytD05; AlytD09; Bir01; Bir04; Jurb02; Kėd01; Kėd01; Kel03; KlaiD07; Mar01; Mol05; Šila01; Šilu10; Šven19; Šven29; Telš03; Telš05; Ute01.

#### *Calamotettix* Emeljanov, 1959

*Calamotettix taeniatus (Horváth, 1911) – AlytD02: 2♂, 1♀, 19 Jul. –03 Aug. 2020, Malaise trap, A. Petrašiūnas leg.; Ign02: 1♀, 21 Jun. 2018, sweep netting, A. Petrašiūnas leg.; 1♀ (MZVUHe01030), 26 Jul. 2020, sweep netting, A. Petrašiūnas leg.; 1♂ (MZVUHe00001), 2♀ (MZVUHe00002, TUZ359701), 23 Jul. 2022, sweep netting, A. Petrašiūnas leg.; Jona01: 2♂, 23 Jul. –07 Aug. 2022, Malaise trap, A. Petrašiūnas leg.; KlaiD07: 1♂, 22 Jul. –05 Aug. 2022, Malaise trap, A. Petrašiūnas leg.; Šiau07: 1 ex (MZVUHe03079), Alkaline fen, 19 Jul. 2024, E. Sriubaite leg.

**Remarks**. A monophagous species on *Phragmites australis* (Nickel 2003). In Northern Europe, it was previously known only from Finland (Söderman et al. 2009; Albrecht et al. 2015). It is also known from the Pskov Region of Russia (Söderman et al. 2009). Since 2012, it has been recorded in Poland (Walczak and Jeziorowska 2015) and since 2011 in Latvia (Ziemelis 2014; Borodin 2023). The species is likely undergoing a northward range expansion.

#### *Cosmotettix* Ribaut, 1942

***Cosmotettix aurantiacus* (Forel, 1859)**: Nast (1987); Della Giustina and Remane (2001); Nickel (2003); Söderman et al. (2009). Laz05 (Vilbaste 1974).

***Cosmotettix costalis* (Fallén, 1826)**: Vilbaste (1974); Nast (1987); Söderman et al. (2009). AlytD02; Anyk01; KlaiD02; Var08.

*Cosmotettix edwardsi (Lindberg, 1924) – KlaiD02: 1♀ (MZVUHe01208), 25 Jun. 2020, sweep netting, A. Petrašiūnas leg.; Šven16: 1♂ (TUZ359779), 28 Jun. 2023, O. Borodin leg.

**Remarks**. A rare species occurring sporadically in Denmark, Sweden, Finland, and Estonia (Nast 1972, 1987; Ossiannilsson 1983; Söderman et al. 2009). It is also known from Canada (Metcalf 1967); Kazakhstan (Emeljanov 1969; Mitjaev 1971, 2002, 2015; Nast 1972; Anufriev and Kirillova 1998); Kyrgyzstan (Dubovsky and Karimov 1970; Nast 1972; Chelpakova 1996; Mitjaev 2015); Latvia (Varzinska 1983; Nast 1987; Spuris 1996); Mongolia (Dworakowska 1973); the European part of Russia (Emeljanov 1964; Tishechkin 1988; Anufriev and Kirillova 1998; Anufriev 2000; Dmitriev 2001; Anufriev and Smirnova 2009); and Uzbekistan (Dubovsky 1966; Mitjaev 2015).

***Cosmotettix panzeri* (Flor, 1861)**: Nast (1987); Söderman et al. (2009). Šven14 (Vilbaste 1974). AlytD02.

#### *Diplocolenus* Ribaut, 1946

***Diplocolenus abdominalis* (Fabricius, 1803)** (= *Verdanus abdominalis* (Fabricius, 1803)): Vilbaste (1974); Nast (1987); Söderman et al. (2009). KauD14 (Ivanauskas et al. 2014b). Ign03; Jurb05; Kal01; KauD04; KlaiD07; Kup05; Mar01; Rok05; Šal01; Šal02; Šven08; Šven19; Šven29; Trak12; Trak13; VilnC01.

***Diplocolenus bohemanni* (Zetterstedt, 1837)**: Vilbaste (1974); Söderman et al. (2009). Zar04.

#### *Errastunus* Ribaut, 1946

***Errastunus ocellaris* (Fallén, 1806)**: Vilbaste (1974); Nast (1987); Söderman et al. (2009). PanC01 (Mensonienė 1979); KauD14 (Ivanauskas et al. 2014b). AlytD01; Ign02; Ign03; Ign09; Jurb05; Kal01; KauD09; Kup01; Kup02; Kup03; Kup05; Mar01; Mol04; Ner02; Ner09; Pag01; Plu03; Rad06; Rok04; Šal01; Šilu04; Šilu05; Šilu09; Šilu10; Šilu11; Šilu12; Šven15; Šven16; Šven17; Šven28; Šven29; Trak12.

#### *Erzaleus* Ribaut, 1952

***Erzaleus metrius* (Flor, 1861)**: Metcalf (1967); Söderman et al. (2009). Bir01 (Vilbaste 1974); Var01 (Söderman and Dapkus 2009). Pag01; Šilu05; Šilu10; Šilu11; Šilu12.

#### *Jassargus* Zakhvatkin, 1933

***Jassargus allobrogicus* (Ribaut, 1936)**: KlaiC01 (Vilbaste 1974). Ner02.

***Jassargus alpinus* (Then, 1896)** (= *J. a. neglectus* (Then, 1896), *J. neglectus* (Then, 1896)): Vilbaste (1974); Söderman et al. (2009). Šven14; Šven17.

***Jassargus distinguendus* (Flor, 1861)** (= *J. pseudocellaris* (Flor, 1861)): Vilbaste (1974); Nast (1987); Söderman et al. (2009). Ner04; PanD02; Šilu02.

***Jassargus flori* (Fieber, 1869)**: Vilbaste (1974); Nast (1987); Söderman et al. (2009). VilnD06 (Ivanauskas et al. 2014b). Ign04; Laz06; Šven03; Šven24; Var36; Zar02.

*Jassargus sursumflexus (Then, 1902) – KlaiD02: 4♂ (MZVUHe01393, MZVUHe01485, MZVUHe01487, MZVUHe01492), 22 Jul. 2022, sweep netting, A. Petrašiūnas leg.; Kel03: 9♂ (MZVUHe02025-MZVUHe02027, MZVUHe02064, MZVUHe02067-MZVUHe02069, MZVUHe02109, MZVUHe02110), 8 ex (MZVUHe03317-MZVUHe03324), 05 Jul. 2024, sweep netting, E. Sriubaite leg.; 51♂ (MZVUHe02011-MZVUHe02024, MZVUHe02033-MZVUHe02043, MZVUHe02055-MZVUHe02062, MZVUHe02078-MZVUHe02083, MZVUHe02096-MZVUHe02107), 31 ex (MZVUHe03370-MZVUHe03400), 19 Jul. 2024, sweep netting, E. Sriubaite leg.; 27♂ (MZVUHe02001-MZVUHe02010, MZVUHe02046-MZVUHe02054, MZVUHe02070-MZVUHe02073, MZVUHe02092-MZVUHe02095), 44 ex (MZVUHe04015-MZVUHe04024, MZVUHe04026-MZVUHe04059), 05 Aug. 2024, sweep netting, E. Sriubaite leg.; 20♂ (MZVUHe02028-MZVUHe02032, MZVUHe02044, MZVUHe02045, MZVUHe02065, MZVUHe02066, MZVUHe02074-MZVUHe02077, MZVUHe02084-MZVUHe02090), 2♀ (MZVUHe02111, MZVUHe02112), 29 ex (MZVUHe04025, MZVUHe04065-MZVUHe04092), 19 Aug. 2024, sweep netting, E. Sriubaite leg.; Šiau07: 2♂ (MZVUHe02124, MZVUHe02125), 6 ex (MZVUHe03311-MZVUHe03316), 05 Jul. 2024, sweep netting, E. Sriubaitė leg.; 11♂ (MZVUHe02091, MZVUHe02113, MZVUHe02114, MZVUHe02116-MZVUHe02123), 45 ex (MZVUHe03325-MZVUHe03369), 19 Jul. 2024, sweep netting, E. Sriubaitė leg.; 1♀ (MZVUHe02063), 14 ex (MZVUHe04001-MZVUHe04014), 06 Aug. 2024, sweep netting, E. Sriubaitė leg.; 2♂ (MZVUHe02108, MZVUHe02115), 5 ex (MZVUHe04060-MZVUHe04064), 19 Aug. 2024, sweep netting, E. Sriubaitė leg.

**Remarks**. A widespread species in Europe (Nast 1972, 1987; Dmitriev et al. 2022), which is monophagous on *Molinia caerulea* (Nickel 2003). It has been recorded in all countries neighbouring Lithuania (Söderman et al. 2009; Borodin 2015b).

#### *Metalimnus* Ribaut, 1948

***Metalimnus formosus* (Boheman, 1845)**: Vilbaste (1974); Nast (1987); Anufriev and Kirillova (1998); Söderman et al. (2009). AlytD09; Anyk01; Anyk02; Šilu12.

***Metalimnus marmoratus* (Flor, 1861)**: Vilbaste (1974); Nast (1987); Söderman et al. (2009). AlytD01; Ign10; Kel05; Mar01; Šila01; Šilu04; Šilu05; Šilu10; Šilu12; Trak03.

***Metalimnus obtusus* Emeljanov, 1966**: Söderman et al. (2009). Var01 (Söderman and Dapkus 2009).

***Metalimnus steini* (Fieber, 1869)**: Söderman et al. (2009). Var01 (Söderman and Dapkus 2009).

#### *Mocuellus* Ribaut, 1946

***Mocuellus collinus* (Boheman, 1850)**: Nast (1987); Vilbaste (1974); Söderman et al. (2009). KlaiD02; Šilu09; Šven14; Trak15.

#### *Paralimnus* Matsumura, 1902

***Paralimnus lugens* (Horváth, 1897)** (= *P. zachvatkini* Emeljanov, 1964): Söderman et al. (2009). Var01 (Söderman and Dapkus 2009).

*Paralimnus phragmitis (Boheman, 1847) – AlytD02: 1♀, 24 Jul. 2020, sweep netting, A. Petrašiūnas leg.; Ign02: 7♂ (MZVUHe00994, MZVUHe01028, MZVUHe01371, MZVUHe01586), 23♀ (MZVUHe00992, MZVUHe00996, MZVUHe00998-MZVUHe01000, MZVUHe01025, MZVUHe01027, MZVUHe01029, MZVUHe01033, MZVUHe01034, MZVUHe01367, MZVUHe01369, MZVUHe01370, MZVUHe01372), 26 Jul. 2020, sweep netting, A. Petrašiūnas leg.; 4♂ (MZVUHe00027, MZVUHe00037, MZVUHe00045, MZVUHe00050), 15♀ (MZVUHe00024-MZVUHe00026, MZVUHe00028, MZVUHe00029, MZVUHe00031-MZVUHe00036, MZVUHe00040, MZVUHe00041, MZVUHe00047, TUZ359712), 23 Jul. 2022, sweep netting, A. Petrašiūnas leg.; Jona01: 1♀ (MZVUHe01352), 23 Jun. 2020, sweep netting, A. Petrašiūnas leg.; 5♂, 2–13 Jul. 2020, Malaise trap, A. Petrašiūnas leg.; 2♀ (MZVUHe01317, MZVUHe01654), 26 Jul. 2020, sweep netting, A. Petrašiūnas leg.; 39♂ (MZVUHe01669, MZVUHe01679), 1♀, 26 Jul. –07 Aug. 2020, Malaise trap, A. Petrašiūnas leg.; 4♂ (MZVUHe00054, MZVUHe00059, MZVUHe00062, MZVUHe00063), 7–23 Jul. 2022, Malaise trap, A. Petrašiūnas leg.; 4♂ (MZVUHe00991), 5♀, 23 Jul. 2022, sweep netting, A. Petrašiūnas leg.; KlaiD07: 1♂, 3♀, 25 Jul. 2020, sweep netting, A. Petrašiūnas leg.; 3♂, 6–22 Jul. 2022, Malaise trap, A. Petrašiūnas leg.; 1♀, 22 Jul. 2022, sweep netting, A. Petrašiūnas leg.; Šiau04: 1 ex (MZVUHe03051), 05 Jul. 2024, E. Sriubaite leg.; 4 ex (MZVUHe03067-MZVUHe03070), 20 Jul. 2024, E. Sriubaite leg.; 1 ex (MZVUHe03077), 19 Aug. 2024, E. Sriubaite leg.; Šiau07: 3 ex (MZVUHe03048-MZVUHe03050), 05 Jul. 2024, E. Sriubaite leg.; 15 ex (MZVUHe03052-MZVUHe03066), 19 Jul. 2024, E. Sriubaite leg.; 6 ex (MZVUHe03071-MZVUHe03076), 06 Aug. 2024, E. Sriubaite leg.; Šilu05: 1♀ (MZVUHe01109), 25 Jul. 2020, sweep netting, A. Petrašiūnas leg.; Šilu10: 3♀, 26 Jul. –06 Aug. 2017, Malaise trap, A. Petrašiūnas, J. Liemke, K. Lingytė leg.

**Remarks**. A widespread species in the western part of the Palearctic region (Dmitriev et al. 2022), but locally rare. Monophagous on *Phragmites australis* (Nickel 2003). Given the species’ distribution pattern, its occurrence in Lithuania appeared highly probable. Previously, only the rare *Paralimnus lugens* (Horváth, 1897) had been reported from Lithuania (Söderman et al. 2009).

#### *Pinumius* Ribaut, 1946

***Pinumius areatus* (Stål, 1858)**: Nast (1987); Anufriev and Kirillova (1998); Söderman et al. (2009); Mitjaev (2015). Šven24 (Vilbaste 1974).

#### *Psammotettix* Haupt, 1929

***Psammotettix alienus* (Dahlbom, 1850)**: Vilbaste (1974); Nast (1987); Söderman et al. (2009). PanC01 (Mensonienė 1979); KauD14 (Ivanauskas et al. 2014b). AlytC01; AlytD04; Kaiš07; Kal02; Kel05; PalM03; Šiau02; Var17; Var37; Vilk01.

***Psammotettix cephalotes* (Herrich-Schäffer, 1834**): Vilbaste (1974); Nast (1987); Söderman et al. (2009); Abdollahi et al. (2015). Telš03.

***Psammotettix confinis* (Dahlbom, 1850)**: Vilbaste (1974); Nast (1987); Söderman et al. (2009). KauD14; VilnD06 (Ivanauskas et al. 2014b). Šilu05; Šven24; Var36; Var37; VilnD07.

***Psammotettix dubius* Ossiannilsson, 1974**: Söderman et al. (2009).

***Psammotettix excisus* (Matsumura, 1906)** (= *P. exilis* Wagner, 1941): Vilbaste (1974); Nast (1987); Nickel (2003); Söderman et al. (2009). Anyk01; Ign06; Ner04; Šven24; Var17; Var37.

***Psammotettix koeleriae* Zakhvatkin, 1948**: Nast (1987); Anufriev and Kirillova (1998); Söderman et al. (2009). Anyk01 (Vilbaste 1974).

***Psammotettix nodosus* (Ribaut, 1925)**: Vilbaste (1974); Nast (1987); Söderman et al. (2009). Šilu02.

***Psammotettix pallidinervis* (Dahlbom, 1850)**: Vilbaste (1974); Nast (1987); Söderman et al. (2009). Ign06; PalM03; Šven24; Var37.

***Psammotettix poecilus* (Flor, 1861)**: Vilbaste (1974); Nast (1987); Söderman et al. (2009). Var37; Zar04.

***Psammotettix sabulicola* (Curtis, 1837)**: Nast (1987); Söderman et al. (2009); Thanou et al. (2018). Ner07; PalC01 (Vilbaste 1974). Ner01; Ner02.

#### *Sorhoanus* Ribaut, 1946

***Sorhoanus assimilis* (Fallén, 1806)**: Vilbaste (1974); Nast (1987); Söderman et al. (2009). KlaiD02; VilnD07.

***Sorhoanus xanthoneurus* (Fieber, 1869)**: Vilbaste (1974); Nast (1987); Söderman et al. (2009). Šven01; Šven06.

#### *Turrutus* Ribaut, 1946

***Turrutus socialis* (Flor, 1861)**: Vilbaste (1974); Nast (1987); Söderman et al. (2009). Anyk01; Ign03; Jurb02; Jurb05; Kaiš06; Kal01; Rad06; Rok05; Šilu04; Šven17; Šven19; Šven25; Šven28; Šven29; Trak12; Zar01.

## Discussion

A total of 360 species belonging to 147 genera have currently been recorded from Lithuania (Table 1); 20 species are reported in this article from Lithuania for the first time, four of which are newly recorded in the Baltic States. A comparison of checklists of Auchenorrhyncha known from countries neighbouring Lithuania suggests that at least 20 additional Auchenorrhyncha species may be discovered in the future. In addition, the potential occurrence of new alien species, including invasive ones, cannot be excluded, as their spread is often facilitated by human activity, primarily through the introduction of ornamental plants.

**Table 1.** Taxonomic structure of the Lithuanian Auchenorrhyncha fauna.

Taxon	Species	Genus	New
Fulgoromorpha Evans, 1946	71	37	11
Delphacoidea Leach, 1815	68	34	11
Cixiidae Spinola, 1839	6	3	0
Delphacidae Leach, 1815	62	31	11
Asiracinae Motschulsky, 1863	1	1	1
Kelisiinae Wagner, 1963	10	1	1
Stenocraninae Wagner, 1963	3	1	2
Delphacinae Leach, 1815	48	28	7
Fulgoroidea Latreille, 1807	3	3	0
Achilidae Stål, 1866	1	1	0
Caliscelidae Amyot & Audinet-Serville, 1843	1	1	0
Tettigometridae Germar, 1821	1	1	0
Cicadomorpha Evans, 1946	289	110	9
Cicadoidea Batsch, 1789	1	1	0
Cicadidae Batsch, 1789	1	1	0
Cercopoidea Leach, 1815	11	5	1
Aphrophoridae Amyot & Audinet-Serville, 1843	11	5	1
Membracoidea Rafinesque, 1815	277	104	8
Membracidae Rafinesque, 1815	3	3	0
Cicadellidae Latreille, 1825	274	101	8
Ulopinae Le Peletier & Audinet-Serville, 1828	2	2	0
Ledrinae Fairmaire, 1855	1	1	0
Megophthalminae Kirkaldy, 1906 [1859]	4	3	0
Eurymelinae Amyot & Audinet-Serville, 1843	36	12	1
Iassinae Walker, 1869	3	2	0
Aphrodinae Haupt, 1927 [1859]	12	4	0
Evacanthinae Crumb, 1911	2	1	0
Cicadellinae Latreille, 1825	1	1	1
Typhlocybinae Kirschbaum, 1868	86	27	1
Deltocephalinae Fieber, 1869	127	48	5
Total	360	147	20

Summary data at the family level are provided in Table 2. Data on Auchenorrhyncha are available for 54 of the 60 municipalities in Lithuania. At present, there are no data from Joniškis District, Pakruojis District, Skuodas District, Šiauliai City, Ukmergė District and Visaginas municipality.

**Table 2.** Species richness of Auchenorrhyncha families across municipalities.

Municipalities	Number of species by family									Total
	Cixiidae	Delphacidae	Achilidae	Caliscelidae	Tettigometridae	Cicadidae	Aphrophoridae	Membracidae	Cicadellidae	
Akmenė District	0	0	0	0	0	0	1	0	1	2
Alytus City	0	0	0	0	0	0	0	1	12	13
Alytus District	0	23	0	0	0	0	6	1	53	83
Anykščiai District	0	7	0	0	0	0	2	1	19	29
Birštonas	0	7	0	0	0	0	1	0	4	12
Biržai District	2	0	0	0	0	0	1	1	19	23
Druskininkai	0	9	0	0	0	0	1	1	3	14
Elektrėnai	1	1	0	0	0	0	0	1	12	15
Ignalina District	3	9	0	0	0	0	6	0	46	64
Jonava District	0	2	0	0	0	0	0	0	4	6
Jurbarkas District	0	2	0	0	0	0	4	0	25	31
Kaišiadorys District	1	1	0	0	0	0	2	2	12	18
Kalvarija	0	4	0	0	0	0	1	0	10	15
Kaunas City	2	0	0	0	1	1	2	1	3	10
Kaunas District	1	4	0	0	0	1	3	1	28	38
Kazlų Rūda	0	4	0	0	0	0	2	1	6	13
Kėdainiai District	0	0	0	0	0	0	0	0	5	5
Kelmė District	0	10	0	0	0	0	4	1	19	34
Klaipeda City	0	0	0	0	0	0	0	1	1	2
Klaipėda District	1	9	0	1	0	0	5	1	23	40
Kretinga District	0	0	0	0	0	0	0	1	2	3
Kupiškis District	0	2	0	0	0	0	1	0	12	15
Lazdijai District	0	4	0	0	0	0	1	2	38	45
Marijampolė	1	3	0	0	0	0	0	0	18	22
Mažeikiai District	0	1	0	0	0	0	0	1		2
Molėtai District	0	1	0	0	0	0	1	0	10	12
Neringa	0	2	0	0	0	0	4	1	19	26
Pagėgiai	0	2	0	0	0	0	0	0	11	13
Palanga City	0	1	0	0	0	0	1	1	6	9
Panevėžys City	1	5	0	0	0	0	4	0	27	37
Panevėžys District	0	5	0	0	0	0	2	0	50	57
Pasvalys District	1	0	0	0	0	0	0	0	4	5
Plungė District	1	0	0	0	0	0	0	1	16	18
Prienai District	0	1	0	0	0	0	1	1	1	4
Radviliškis District	1	0	0	0	0	0	0	1	6	8
Raseiniai District	0	1	0	0	0	0	1	0	19	21
Rietavas	0	0	0	0	0	0	0	0	1	1
Rokiškis District	0	2	0	0	0	0	5	0	20	27
Šakiai District	3	1	0	0	0	0	4	2	11	21
Šalčininkai District	0	3	0	0	0	0	0	1	5	9
Šiauliai District	2	9	0	0	0	0	5	1	16	33
Šilalė District	0	1	0	0	0	0	0	0	8	9
Šilutė District	2	11	0	1	0	0	4	0	56	74
Širvintos District	0	0	0	0	0	0	1	1	2	4
Švenčionys District	3	12	0	1	0	0	5	2	84	107
Tauragė District	1	1	1	0	0	0	1	0	8	12
Telšiai District	0	4	0	0	0	0	0	1	16	21
Trakai District	1	4	0	0	0	0	1	1	57	64
Utena District	1	0	0	0	0	0	4	1	18	24
Varėna District	3	8	1	0	0	0	7	2	62	83
Vilkaviškis District	0	2	0	0	0	0	1	0	31	34
Vilnius City	2	2	0	0	0	0	0	1	7	12
Vilnius District	2	6	0	0	1	0	2	0	39	50
Zarasai District	1	1	0	0	0	0	1	0	11	14
Total	6	62	1	1	1	1	11	3	274	360

As shown in Table 2, relatively few species have been recorded in most municipalities, indicating that the Auchenorrhyncha fauna remains insufficiently studied at the regional level. The highest numbers of species were recorded in the municipalities of Švenčionys District (107 species), Alytus District (83 species), and Varėna District (83 species). Thus, as a result of summarizing all information available at the time of this study, occurrence localities are provided for 338 species, and additional, previously unpublished data are presented for 275 species. It should also be noted that a considerable portion of the data was obtained from processing materials collected within monitoring programs in which Auchenorrhyncha were not the target group. This situation is analogous to that often encountered in ichthyology, where faunistically significant findings of non-target species regularly occur as bycatch during commercial fishing (Werner et al. 2015; Jones et al. 2018; Villafáfila et al. 2025).

A system should therefore be developed to ensure the preservation of all material collected by various types of traps during monitoring studies, regardless of focal taxa. This would increase the scientific value of collections that contain such material. Attention should also be paid to the preservation of historical collections, which enable tracing, from a historical perspective, the approximate time of appearance of new species in the local fauna, including those that have entered the region naturally through range expansion. A clear example is *Calamotettix taeniatus*, a reasonably common species at present, which J. Vilbaste did not record despite more than a decade of research on the Lithuanian and Latvian leafhopper fauna. This suggests that the species colonised the region relatively recently, rather than being rare or difficult to detect. At the same time, the absence of records of *Cicadetta montana* s.str. in the 1960s–1970s in Latvia, Lithuania, and Estonia, can only be explained by an apparent local distribution of this species’ populations and its specific biological traits.

## Additional information

### Use of AI

No use of AI was reported.

### Author ORCIDs

Oleg Borodin https://orcid.org/0000-0001-8907-2139

Andrius Petrašiūnas https://orcid.org/0000-0003-3270-3694

Vytautas Tamutis https://orcid.org/0000-0002-6120-2066

Villu Soon https://orcid.org/0000-0001-5368-0319

### Data availability

All of the data that support the findings of this study are available in the main text.
